# Recent Advances in the Synthesis and Biological Applications of Prenylated Chalcones

**DOI:** 10.3390/ijms26209845

**Published:** 2025-10-10

**Authors:** Mouna Hind Laiche, James W. Barlow

**Affiliations:** Department of Chemistry, RCSI University of Medicine and Health Sciences, 123 St. Stephen’s Green, D02 YN77 Dublin, Ireland; mounahindlaiche22@rcsi.ie

**Keywords:** prenylation, chalcone, synthesis, biological activity

## Abstract

Prenylated chalcones, a subclass of chalcones distinguished by the addition of one or more prenyl (3-methylbut-2-enyl) groups, have attracted significant attention due to their promising biological activities. The origins, chemical diversity, and synthetic routes used to prepare naturally occurring and synthetic prenylated chalcones are discussed in this review paper, alongside their diverse pharmacological properties, as reported over the past 10 years (2015–2025), mainly emphasising their strong anti-cancer, anti-inflammatory, anti-bacterial, anti-fungal, anti-parasitic, and anti-malarial effects. We address their structure–activity relationships (SARs) to interrogate how prenylation affects the pharmacological activity of these chalcones.

## 1. Introduction

Chalcones are a diverse group of natural products, occurring in both fungal and plant kingdoms, including within several species traditionally used in folk medicine [1]. They have attracted considerable attention due to their remarkable pharmacological activities, encompassing antioxidant, anti-cancer, anti-diabetic, anti-inflammatory, neuroprotective, cardioprotective, and anti-microbial properties, and therefore have many potential therapeutic applications [2]. The core of all chalcones is the 1,3-diaryl-2-propen-1-one unit **1**, template of the open-chain flavonoids [3], with both aryl rings (A-ring, the acetophenone component and B-ring, the aldehyde component) substituted with different functional groups, but typically having one or more phenolic functionalities [4]. Much of the reactivity and biological effects of chalcones are associated with the electrophilic α, β-unsaturated carbonyl system, yet distinct properties are also exhibited by various sub-classes. These include their reduced derivatives, the dihydrochalcones, and further, chalcone dimers (bischalcones) and various glycoside derivatives.

**Figure 1:**
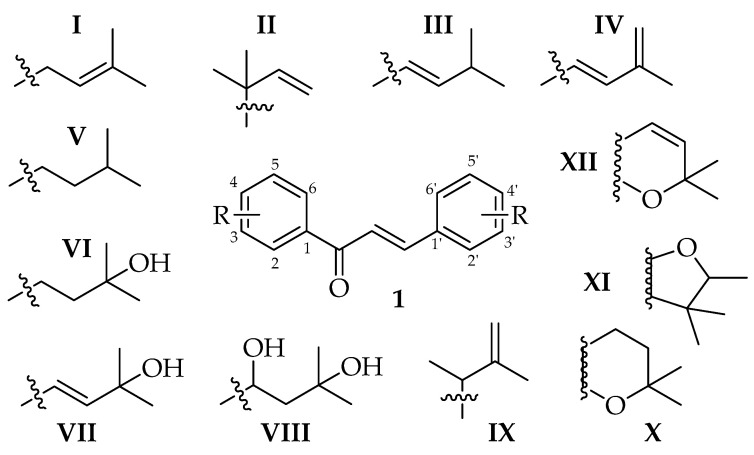
Prenylation patterns (C5 groups among R variants) found within prenylated chalcones.

Additional complexity may be introduced into chalcone skeletons by the process of prenylation, which signifies the introduction of at least one 3-methylbut-2-enyl or isopentenyl unit. The variation in number, saturation, and branching of prenyl units (usually C5, C10, or C15 chains), their positioning, and their substitution or cyclisation patterns (see Figure 1 for C5 variants, I-XII) on the chalcone structure allows for significant molecular diversity [3]. The presence of prenyl units can therefore have a significant impact on physicochemical, pharmacological, and pharmacokinetic parameters. The impact of prenylation is significant and encountered across many natural products as a mechanism of diversification, introducing the complexity of structure and function to diverse alkaloids and phenylpropanoids, the latter including acetophenones, alcohols and esters, aldehydes, anthraquinones, benzoic acids, benzophenones, cinnamic acids, coumarins, flavonoids, lignans, terphenyl derivatives, xanthones and chalcones [5]. Prenylation has been repeatedly shown to increase activity compared to the non-prenylated analogue [6,7]. This phenomenon has been attributed to the fact that prenylation may increase interactions with target proteins and biological membranes, increasing the bioavailability and effectiveness of such compounds [8]. The prenyl moiety contributes to a stronger membrane affinity, facilitating better cellular absorption and interaction with biomolecular targets, including enzymes and receptors [9].

However, prenylated secondary metabolites typically occur in their sources at low levels, hindering their isolation and further study. Advances in extraction have however facilitated studies into the mechanisms of action and therapeutic potential of these compounds [10]. Technological developments in analysis have also made it easier to distinguish and characterise chalcones from related compounds in intricate plant matrices [11]. Additionally, synthetic protocols to prenylated chalcones have evolved in the recent past, allowing for molecular diversification [12]. Relevant methodologies include enzymatic prenylation and chemical reactions including the Williamson etherification, Friedel–Craft acylations and sigmatropic rearrangements, which have been utilised to selectively add and rearrange prenyl groups, expanding the range of bioactive chalcone derivatives that can be investigated [13]. Thus, a review of these methodologies and the activity of the resulting molecules is timely. Also, while many existing publications focus on the diverse applications of simpler chalcones, reviews with a more specific focus on prenylated chalcones remain scarce. Zhou and collaborators [3] previously conducted a comprehensive review on naturally occurring prenylated chalcones. In addition, Singh and collaborators [14] recently published a tailored review on the synthesis and biological activity of chromenochalcones, which are a heterocyclic division (Figure 1, XII) of the prenylated chalcones. To reflect the gaps in the existing literature, in this paper, our focus was to more broadly collate the many structural features associated with prenylated chalcones, with a particular focus on synthetic routes to their preparation and diversification to optimise biological activity and how these developments might inform chalcone-based drug development in the future [3,15].

To extract relevant papers on the synthesis of prenylated chalcones, we utilised three electronic databases (PubMED, Elsevier’s Scopus, and Google Scholar). All papers published up to September 2025 were included, covering the total synthesis of natural prenylated chalcones and their synthetic derivatives. To interrogate biological activity, we focused on research papers from January 2015 to September 2025, using the same three electronic databases and limiting the analysis to the first 20 pages (when relevant) and to articles written in English. Search terms were utilised in several combinations, including ‘prenylated chalcones synthesis’ and ‘biological activity of prenylated chalcones’, with an emphasis on xanthohumol and Licochalcone A.

## 2. Clinical Application of Prenylated Chalcones

**Figure 2:**
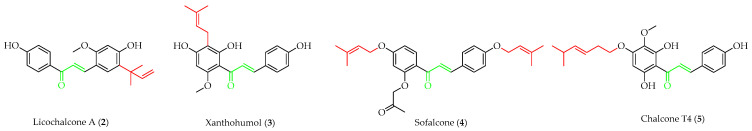
Notable examples of prenylated chalcones (2–5) investigated in clinic.

Prenylated chalcones hold promise as lead molecules [16], with notable examples having advanced through various stages of drug development (Figure 2).

Licochalcone A (**2**), isolated from Chinese liquorice (*Glycyrrhiza glabra*), is a notable prenylated chalcone [17], having been evaluated in clinic for dermatological applications. When combined with 1,2-decanediol, L-carnitine, and either salicylic acid [18] or adapalene [19], reductions in acne lesion counts were observed. As an anti-irritant, when included as an ingredient in a skin care regimen for patients with mild-to-moderate facial redness, the formulation was compatible with sensitive facial skin in rosacea patients and enhanced the appearance of persistent facial redness. Additionally, the products were compatible with daily metronidazole treatment [20]. It (**2**) has also undergone early clinical evaluation as a photoprotective treatment alongside adapalene in the treatment of acne, whereby it reduced the dryness, burning, and stinging associated with the retinoid [21].

Another important prenylated chalcone which has also been evaluated in clinic is xanthohumol (**3**), which is isolated from the female inflorescences of hop cones (*Humulus lupulus*). Among 15 registered trials, with a broad focus on viral, immunological, and metabolic properties, of specific interest was a study on the effects of **3** as an adjuvant treatment for patients admitted to the ICU due to COVID-related acute respiratory failure [22]. While the reported preliminary findings suggested that **3** improved the clinical course of patients and reduced both the severity of the inflammatory response and mortality rate, the study was limited by recruitment numbers, and an extended trial is currently suspended. Another study at the same centre is currently recruiting participants in an evaluation of **3** as a supportive therapy in patients with septic shock [23].

In addition to trials of natural products, the synthetic prenylated chalcone sofalcone (**4**), a more stable synthetic analogue of sophoradin, a chalcone isolated from *Sophora subprostrata* [24], has demonstrated gastroprotective properties. It (**4**) promoted gastric ulcer healing during 7 weeks of treatment following 1 week of *H. pylori* eradication therapy, with a healing rate equivalent to that of cimetidine. A clinical trial of the synthetic geranylated chalcone T4 (**5**) has started recruitment to evaluate the effects of local application of **5** on periodontitis and will be evaluated using clinical indices of bleeding on probing, clinical attachment level, and gingival recession, alongside microbiological profiling and the evaluation of inflammatory makers [25].

Apart from studies in clinic such as these, many diverse prenylated chalcones show promising effects, albeit at earlier stages of development, and will be discussed in Section 6.

## 3. Naturally Occurring Prenylated Chalcones

Naturally occurring prenylated chalcones are mostly found in the plant kingdom, particularly within the *Leguminosae*, *Moraceae*, *Cannabaceae*, and *Fabaceae* families [3], including as exemplar genera *Dorstenia*, *Sophora*, *Humulus* [26], and *Glycyrrhiza* [27]. Numerous biological activities and physicochemical characteristics are influenced by the structural differences among these compounds [27]. This structural variety influences how they interact with biological targets [3] and contributes to their anti-bacterial [28], anti-inflammatory [29], antioxidant [30], and anti-cancer effects [8]. This variety may reflect the diversity of the prenylated chalcones’ ecological role in nature [31].

Up until July 2020, Zhou and collaborators [3] comprehensively covered 250 naturally occurring prenylated chalcones, while in Table 1, we have listed more recently isolated novel prenylated chalcones from July 2020 until now. Among these compounds, **8** and **9** were tested against SARS-CoV-2, and **11** was tested for anti-proliferative effects; however, none of the compounds showed any activity.

## 4. Biosynthesis of Prenylated Chalcones

Prenylated chalcones originate through the addition of prenyl groups to already synthesised chalcone cores during the biosynthetic process [3]. The polyketide pathway, which produces aromatic polyketide intermediates through the activity of polyketide synthases, is typically where biosynthesis starts with the creation of a basic chalcone skeleton [38]. Prenyltransferases catalyse the transfer of prenyl groups, such as dimethylallyl or geranyl units, from prenyl diphosphate donors to specific sites on the aromatic rings of chalcones once the central 1,3-diaryl-2-propen-1-one core is created [39]. The term ‘prenyltransferase’ generally refers to enzymes that facilitate the transfer of prenyl groups to various acceptors, including isopentenyl diphosphate (IPP), aromatic compounds, proteins, and others [40]. The biological and chemical diversity of chalcones is greatly enhanced by this prenylation phase, which contributes to their involvement in pigmentation, plant defence, and possible medicinal uses [41]. The entire process takes place inside specialised plant tissues or cells such as glandular trichomes [42] and is strictly regulated. It frequently reacts to cues from the environment or developmental stages [43].

Recently, attempts have been made to utilise isolated prenyltransferases to elaborate a range of phenolic substrates, including chalcones. Prenylations of ten chalcones (**13**–**22**) were performed using two fungal prenyltransferases (*Aspergillus niger* aromatic prenyltransferase (AnaPT) and *Aspergillus terreus* aromatic prenyltransferase (AtaPT)) in the presence of dimethylallyl diphosphate, and eleven mono-prenylated and four di-prenylated products (**23**–**37**) were obtained (Table 2) [15].

Using a novel recombinant dimethylallyl tryptophan (DMAT) synthase from the thermophilic fungus, *Rasamsonia emersonii,* a number of substrates were subject to successful *O*-prenylation, with a single chalcone (isoliquiritigenin, **22**) undergoing between 1 and 10% conversion [44]. The geranylation of eight chalcones was achieved (Table 3) using AtaPT [39]. Together, these studies show the potential of prenyl transfer in biomimetic approaches to novel chalcone variants.

## 5. Total Synthesis of Prenylated Chalcones

The key step in the classical synthesis of chalcones typically relies on the elaboration of appropriate acetophenone and benzaldehyde synthons, with their subsequent aldol condensation. Methodologies to obtain prenylated chalcones and their derivatives involve either the initial prenylation of one or both aromatic rings (acetophenone or benzaldehyde) prior to condensation, or the prenylation of the already formed chalcone backbone.

### 5.1. O-Prenylation

#### 5.1.1. *O*-Prenylation in Alkaline Media

**Scheme 1:**
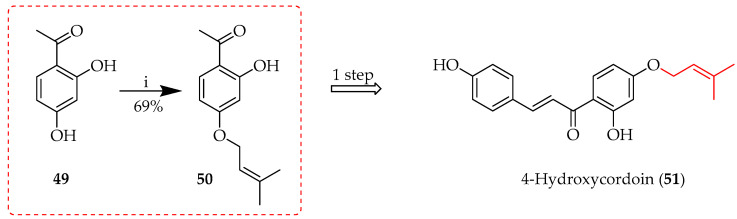
*O*-prenylation of **49** in alkaline media. Reagents and conditions: (i) Prenyl bromide, K_2_CO_3_, acetone, reflux, and 3 h.

Although simple prenylation of a phenoxide generated under basic conditions is a well-established and trivial transformation, judicious choice of reaction conditions can have a significant effect on the outcome and yield. The prenyl group may be added to either the chalcone backbone or to an aromatic precursor prior to condensation. Carbonate bases are often the first choice to deprotonate the phenolic substrate, increasing its nucleophilicity and allowing for a more effective attack of prenyl halides. In addition to enhancing the nucleophilic reactivity, the alkaline conditions aid in transition state stabilisation, raising the overall efficiency of the reaction. This reaction is typified by the initial 4-*O*-prenylation of **49** in the first total synthesis of 4-hydroxycordoin (**51**) [45,46] (Scheme 1).

Prenyl bromide is the most widely used commercial prenylating agent, being more reactive in alkylation due to its better leaving group (bromide) than prenyl chloride, which is less reactive and may require stronger conditions to accomplish comparable levels of alkylation. The choice between the two is determined by a given reaction’s unique requirements and the sensitivity of the substrates in question. Along with alkali carbonates, a wide range of bases can catalyse *O*-prenylation. Each base has differential effects on the yield and efficiency of the reaction and can affect the final product profile. A comparison of various bases employed in *O*-prenylation is shown in Table 4, along with associated yields. For ketone synthon **50**, the amidine base 1,8-diazabicyclo [5.4.0]undec-7-ene (DBU) proved more efficient than hydroxide, while in preparation of aldehyde **53**, the hydride base resulted in high yields of product. Such findings provide insight into how base selection affects reaction outcomes and can be used to optimise conditions for improved yields and increased efficiency in synthetic applications.

#### 5.1.2. The Mitsunobu Reaction

**Scheme 2:**
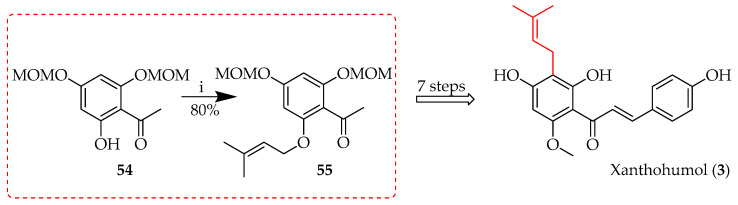
Mitsunobu reaction of **54**. Reagents and conditions: (i) 3-Methyl-2-butene-1-ol, diethylazodicarboxylate, PPh_3_, toluene/tetrahydrofuran (THF), RT, and 12 h. MOM = methoxymethyl.

The Mitsunobu reaction employs a phosphine reagent and an azodicarboxylate to convert alcohols into good leaving groups for nucleophilic substitution [50]. In prenylation, this reaction efficiently introduces prenyl groups to substrates under mild conditions, crucial for modifying natural products and synthesising bioactive molecules. The reaction’s stereospecificity ensures precise control, enhancing the biological activity and solubility of the resulting compounds. During the total synthesis of **3** (Scheme 2) [51], the prenylation of acetophenone **54** using Mitsunobu conditions yielded 80% of intermediate **55**.

#### 5.1.3. Catalytic *O*-Prenylation

The use of transition metals in aryl *C−O* cross-coupling processes has greatly impacted the synthesis of novel compounds [52], such as the coupling of the primary alcohol 3-methylbut-2-en-1-ol with aryl halides **56**–**58** in the presence of a catalyst. Table 5 shows explored reaction variables using nickel under thermal conditions alongside PhSiH_3_ as a reducing agent combined with NiBr_2_-bipyridine and, without relying on photocatalysis or electrocatalysis, produces in situ active Ni(I) intermediates that effectively catalyse the synthesis of aryl *C–O* bonds through a Ni(I/III) cycle [52]. Variation in the halide substrate did not affect the yield of **59** (entry 1 vs. 2). On the other hand, copper-catalysed prenylation of both bromo and iodo intermediates was achieved in the presence of 1,10-phenanthroline [53], with higher yields for the iodo **58** than bromo **57**, offering a comparable yield to nickel-catalysed prenylation (entry 3 vs. 4).

### 5.2. C-Prenylation

#### 5.2.1. Direct Prenylation Strategies

##### *C*-Prenylation in Alkaline Media

**Scheme 3:**
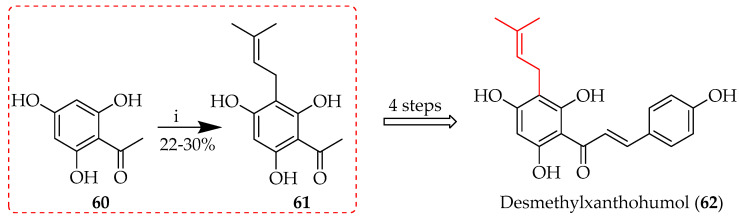
*C*-prenylation of **60** in alkaline media. Reagents and conditions: (i) Prenyl bromide, KOH, H_2_O, RT, and 2 h.

While the use of hydroxide bases usually facilitates *O*-alkylation, in some cases, direct *C*-alkylation is observed. In the total synthesis of desmethylxanthohumol (**62**), *C*-prenylation of **60** (Scheme 3) occurred in an aqueous alkaline medium in the presence of prenyl bromide, albeit in a relatively low yield, producing 22–30% of **61** [54].

**Scheme 4:**
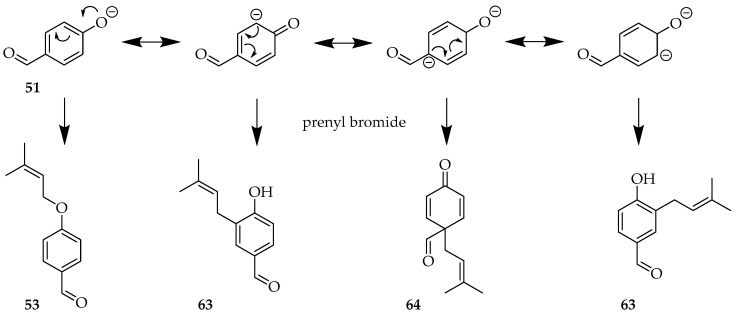
Phenoxide delocalisation during prenylation of **52** leads to loss of regioselectivity.

**Scheme 5:**
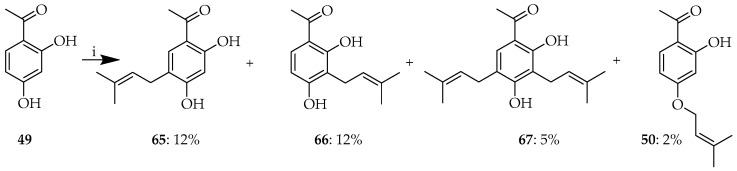
(i) Product profile upon prenylation of **49**. Reagents and conditions: (i) Prenyl bromide, KOH, H_2_O, RT, and 12 h.

Several reports regarding setbacks and low yields in such phenol *C*-alkylation reactions provide a mechanistic explanation: the phenoxide anion delocalises within the aromatic ring (Scheme 4) [55], resulting in a spectrum of different intermediates that explain the mixture of prenylated compounds, including both *C*- and *O*-prenylated products. This is further illustrated by the prenylation of **49** (Scheme 5) [49].

As with *O*-prenylation, various bases can be used for *C*-prenylation. This can impact reaction outcomes, including product yield(s) and the stability of intermediates. Table 6 lists the impact of base choice on the *C*-prenylation of benzaldehydes and acetophenones. Among the reactions of benzaldehyde **68**, metal oxides favoured *C*-3 prenylation, while hydroxides favoured a reaction at the *C*-5 position. In all cases, yields were low, with potassium hydroxide-mediated prenylation of **68** achieving a 30% yield. With acetophenone **70**, the use of amidine base DBU led to a yield of 38% of *C*-3 prenylated **74**.

##### Palladium-Catalysed Prenylation

Various Pd-catalysed cross-coupling strategies have been employed in the elaboration of prenylated chalcones, notably Heck, Suzuki, and Stille couplings. The Heck reaction is a palladium-catalysed coupling of an aryl halide with an alkene to form a substituted alkene. The Stille coupling features the reaction of an organostannane with a suitable electrophile, while Suzuki (or Suzuki–Miyaura) coupling combines an organic halide or pseudohalide with an organoboron reagent (such as an aryl or vinyl boronic acid) [59]. These reactions form new carbon–carbon bonds through oxidative addition, transmetalation, and reductive elimination stages in the presence of a base.

**Scheme 6:**
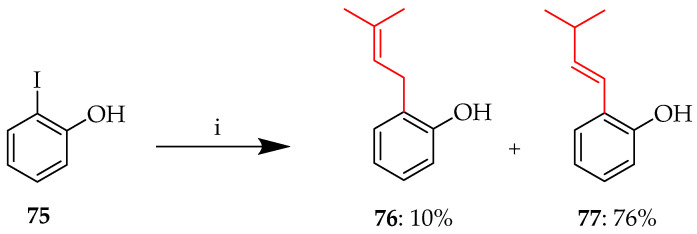
*C*-prenylation of **75**. Reagents and conditions: (i) 3-Methyl-1-butene, Pd(OAc)_2_, Et_3_N, 100 °C, and 20 h.

The Heck reaction requires a base, a palladium catalyst, and usually an aryl iodide or bromide. *C*-prenylation of 2-iodophenol **75** was performed using Heck conditions, with Pd(OAc)_2_ as the palladium catalyst and triethylamine as the base in DMF, to afford two different prenylated phenols: **76** was obtained in 10% yield while the major product, **77**, was obtained in a yield of 76% (Scheme 6) [49].

**Scheme 7:**
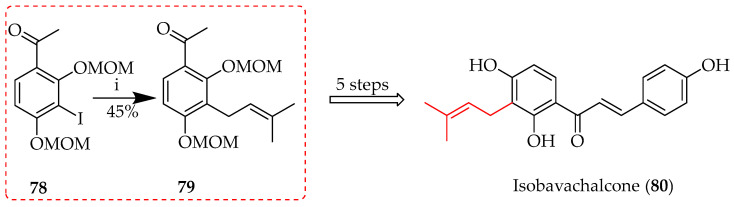
Stille coupling of **78**. Reagents and conditions: (i) Pd(PPh_3_)_4_, prenyltributylstannane, DMF, 100 °C, and 40 h.

Similarly, using ketone precursor **78**, a key intermediate **79** towards isobavachalcone (**80**) (Scheme 7) was obtained in a 45% yield following the palladium-catalysed Stille coupling of **78** using prenyltributylstannane [49].

**Scheme 8:**
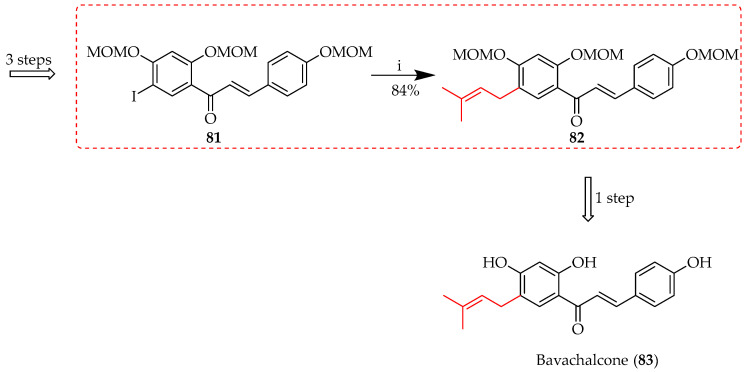
Suzuki coupling of **81.** Reagents and conditions: (i) 3-Methyl-2-butenylboronic acid pinacol ester, PdCl_2_(dppf), Cs_2_CO_3_, DMF, 70 °C, and 2 h.

Chalcone **83** was synthesised via a Suzuki coupling approach, using 3-methyl-2-butenylboronic acid pinacol ester in the prenylation of **81** (Scheme 8) [60,61].

The impact of the choice of the base and catalyst for this illustrative Suzuki coupling is shown in Table 7, with yields ranging from 25 to 84% [60].

##### Friedel–Crafts Prenylation

**Scheme 9:**
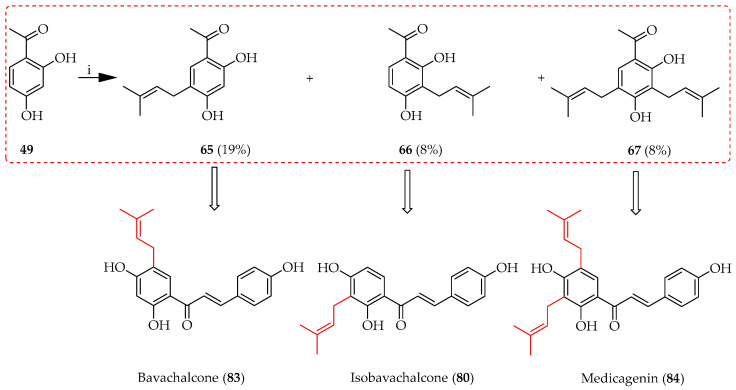
Friedel–Crafts alkylation of **49**. Reagents and conditions: (i) 3-Hydroxy-3-methyl-1-butene, BF_3_.OEt_2_, dioxane, RT, and 5 h.

Friedel–Crafts prenylation involves the alkylation of an aromatic ring with a prenyl moiety mediated by a Lewis acid catalyst, such as aluminium chloride (AlCl_3_) or boron trifluoride (BF_3_). Activation of the electrophile (alcohol substrate) utilised as the prenylation agent makes it more reactive towards the aromatic ring by accepting a pair of electrons. Upon the generation of a carbocation intermediate, this electrophilically attacks the aromatic ring. To prevent side reactions like polyalkylation and rearrangement, the process needs to be carefully regulated. The method is illustrated in the first total synthesis of three prenylated chalcones, bavachalcone (**83**) [62], isobavachalcone (**80**) [49], and medicagenin (**84**) [63], in yields of 8–19% (Scheme 9) using **49** as the starting material for all three. Upon reaction, **49** affords two monoprenylated (C5 and C3) and one diprenylated (C3,5) intermediate. Despite the lack of regioselectivity seen, in this case, all three intermediates were synthetically valuable.

**Scheme 10:**
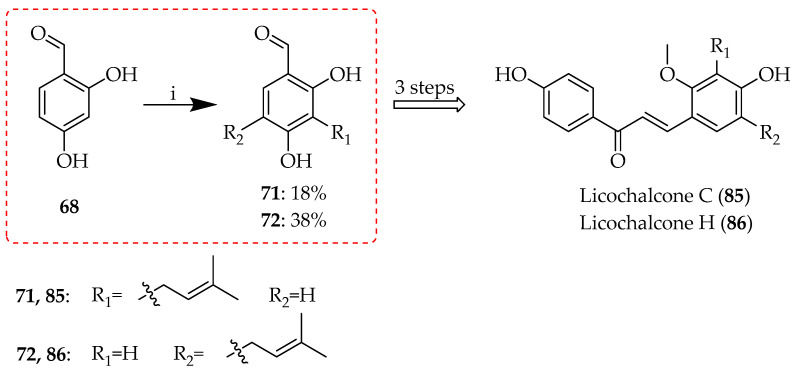
Friedel–Crafts alkylation of **68**. Reagents and conditions: (i) 3-Hydroxy-3-methyl-1-butene, BF_3_.OEt_2_, dioxane, RT, and 5 h.

The reaction has also been applied to aldehyde precursors, exemplified by the reaction of **68** during the synthesis of licochalcones C and H (Scheme 10) [64], offering a higher yield compared to the reaction on the corresponding acetophenones (Scheme 9), likely due to the higher reactivity of the benzaldehyde, alongside less steric hindrance. Again, in this case, both regioisomeric alkylation products **71** and **72** were useful.

#### 5.2.2. Rearrangements of Prenylated Substituents

##### Prenyl Migration Without Isomerisation

Sigmatropic rearrangements are pericyclic reactions in which a sigma bond appears to move across a conjugated system to a new site. These rearrangements usually happen through a coordinated process that involves a cyclic transition state and preserves orbital symmetry. Both heat and photochemical conditions can drive the reaction, with the latter frequently enabling otherwise unattainable rearrangements. The nomenclature refers to the atoms involved: [1,5]-sigmatropic rearrangement occurs when pi-electrons are simultaneously rearranged alongside sigma bond migration between the first and fifth atoms, as shown by the notation “1,5”. Though less frequent than its [1,5] counterpart, the [1,3]-sigmatropic shift is also essential to the rearrangement of molecular frameworks and has enabled the synthesis of sophisticated materials and complicated natural products.

**Scheme 11:**
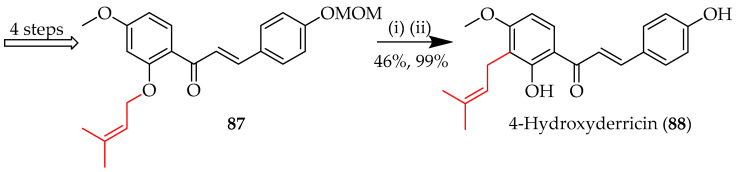
The [1,3] sigmatropic rearrangement of **87**. Reagents and conditions: (i) Montmorillonite K10, DCM, 0 °C, and 1.5 h; (ii) *p*-TsOH–H_2_O, MeOH, 30 °C, and 24 h. DCM = dichloromethane.

During the total synthesis of 4-hydroxyderricin (**88**) [65], prenyl migration to the *ortho* carbon C3 was achieved in a 46% yield via the clay-catalysed [1,3] sigmatropic rearrangement of synthon **87**, with MOM deprotection using *para*-toluenesulfonic acid (*p*-TsOH) to afford the final product (Scheme 11).

In contrast, the treatment of *O*-3,3-dimethylallyl acetophenone synthon **90** with Montmorillonite K10 or Florisil afforded three different *C*-3,3-dimethylallyl products (**91**–**93**; Table 8) [65]. In the presence of Montmorillonite K10, the *para* rearrangement product **91**, following prenyl migration to C5, was isolated in a higher yield than the *ortho* isomer **92** (53 vs. 25%). With Florisil, *para* and *ortho* isomers were isolated in similar yields (27 vs. 32%).

**Scheme 12:**
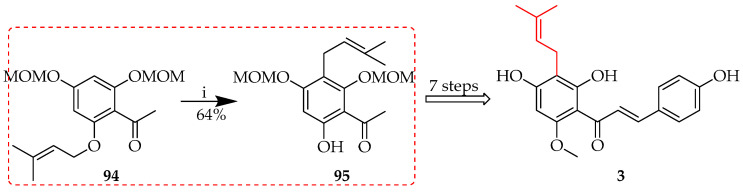
Cope rearrangement of **94**. Reagents and conditions: (i) *N*,*N*-Dimethylaniline, reflux (200 °C), and 6 h.

Rearrangement to the *para* position represents a variant of the Cope rearrangement, a sigmatropic reaction that involves the coordinated remodelling of a 1,5-diene structure, in which the central sigma bond breaks and two new sigma bonds form, causing the double bonds to shift positions. This reaction takes place in a six-membered cyclic transition state with a six-electron suprafacial shift. It is thermally permissible, frequently requiring heat, and can be affected by the presence of substituents on the diene. During the synthesis of xanthohumol (**3**) (Scheme 12), the heating of prenylated ketone precursor **94** facilitated the rearrangement of the prenyl to the *para* position, affording the protected intermediate **95** in a 64% yield [51].

**Scheme 13:**
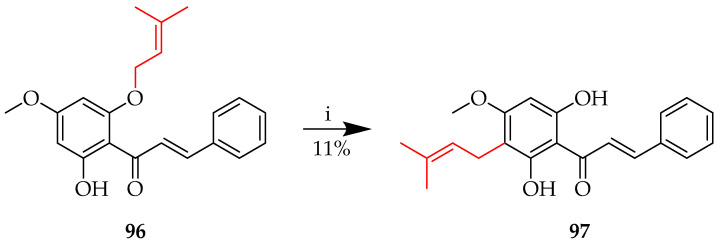
Cope rearrangement of **96**. Reagents and conditions: (i) ZnCl_2_, toluene, reflux, and 24 h.

As with the rearrangement of **90** described above, a range of products was also isolated following the reaction of **96** in the presence of ZnCl_2_ in toluene with reflux (Scheme 13). Among these was **97**, following the rearrangement of prenyl to the *para* position. In contrast to Scheme 12, this analogous reaction product was isolated in only 11% yield, highlighting the importance of a judicious choice of reaction conditions [66].

##### Prenyl Migration with Isomerisation

The Claisen rearrangement, a [3,3]-sigmatropic rearrangement whereby three atoms on either side of the migrating bond change places, is an important transformation in the reactions of prenylated chalcones. Licochalcone A (**2**) (Scheme 13) was synthesised through a [3,3] sigmatropic rearrangement under microwave (MW) conditions, whereby irradiation of the *O*-(3-methylbut-2-en-1-yl) intermediate **98** afforded the *C*-(2-methylbut-3-en-2-yl) system in **2** [67]. Various conditions screened are listed in Table 9, along with their corresponding yields. Among these, microwave irradiation (entry 1) proved most efficient in promoting the desired [3,3]-sigmatropic rearrangement.

**Scheme 14:**
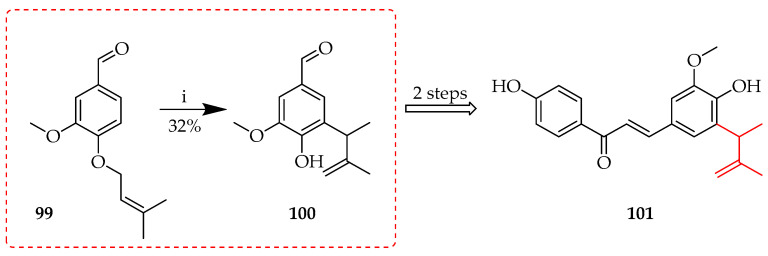
Abnormal Claisen rearrangement of **99**. Reagents and conditions: (i) *N*,*N*-Dimethylaniline, 185 °C, and 26 h.

In contrast, an ‘abnormal’ Claisen rearrangement occurred upon thermal treatment of the *O*-(3-methylbut-2-en-1-yl) aldehyde precursor **99** in *N,N*-dimethylaniline to afford the *C*-(3-methylbut-3-en-2-yl) rearrangement product 99, a key intermediate towards **101** (Scheme 14) [68].

**Scheme 15:**
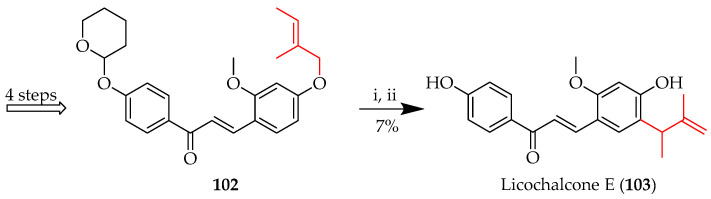
Claisen rearrangement of **102**. Reagents and conditions: (i) *N*,*N*-Dimethylaniline, reflux, 4 h; (ii) 1M HCl, EtOH, RT, and 1 h.

Similar thermal conditions were also used to induce the [3,3]-sigmatropic rearrangement of **102** towards licochalcone E (**103**) (Scheme 15) [69], whereby the *O*-(2-methylbut-2-en-1-yl) system in **102** was rearranged to a *C*-(3-methylbut-3-en-2-yl) unit, with consequent acid-mediated deprotection to reveal **103**. In both these cases, *ortho* rearrangement of the prenyl occurred as the *para* position was already occupied.

**Scheme 16:**
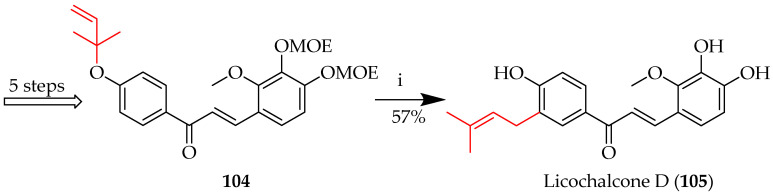
Claisen rearrangement of **104**. Reagents and conditions: (i) EtOH/H_2_O (4/1, *v*/*v*), 120 °C-bomb reactor, and 15 h. MOE = methoxyethyl.

To generate the high temperatures and pressures necessary for these reactions to proceed efficiently, a bomb reactor may be used. This can improve reaction rates and yields by maintaining solvents in the liquid state while above their boiling temperatures, particularly when conventional laboratory conditions are insufficient. It is therefore a useful instrument for carrying out such high-temperature organic reactions. This approach is illustrated by the first synthesis of licochalcone D (**105**) (Scheme 16) [70], whereby the *O*-(2-methylbut-3-en-2-yl) of **104** was rearranged to a *C*-(3-methylbut-2-en-1-yl), with consequent acid-mediated deprotection to reveal **105**.

##### *C*-Prenyl Modification Without Migration

Under certain conditions, a molecule containing a prenyl group may be transformed via *C*-prenyl rearrangement but without a positional shift. This involves adjustments to the bond arrangements or the location of a double bond within the prenyl moiety. In order to prevent the prenyl group from migrating, and to maintain the structural integrity of the target molecule, reaction conditions such as the solvent, temperature, and catalyst(s) must be carefully chosen.

**Scheme 17:**
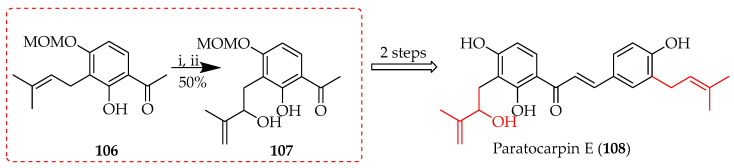
Rearrangement of **106**. Reagents and conditions: (i) *meso*-TPP, O_2_, *hv* (500W), DCM, 5 °C, 12h; (ii) Ph_3_P, RT, and 3 h.

The Schenck ene reaction is a photochemical process in which singlet oxygen (^1^O_2_) reacts with an alkene to form an allylic hydroperoxide. Singlet oxygen may be generated through the exposure of molecular oxygen (O_2_) to light to activate it into its excited singlet form. Typically, this is performed with the aid of a photosensitiser [71] such as tetraphenylporphyin (TPP). The reaction of singlet oxygen with the alkene simultaneously targets the double bond and the allylic hydrogen in a coordinated manner [72]. This reaction is useful for controlled oxygenation, as it proceeds stereospecifically and without intermediates [73]. The reaction was used during the synthesis of paratocapin E (**108**) through the rearrangement of the *C*-(3-methylbut-2-en-1-yl) in **106** to the *C*-(2-methylbut-1-en-3-ol) in **107** (Scheme 17) [74].

**Scheme 18:**
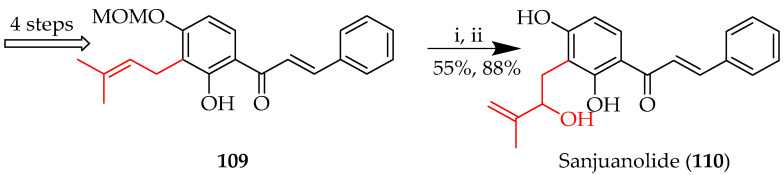
Schenck ene reaction of **109**. Reagents and conditions: (i) TPP, DCM, *hv*, O_2_, 10 °C, and 4 h; PPh_3_, RT, and 5 h; and (ii) *p*-TsOH·H_2_O, THF/MeOH, 50 °C, and 15 h.

The Schenck ene reaction is further exemplified by the total synthesis of sanjuanolide (**110**) (Scheme 18) [75], which involved the photooxidation of **109** bearing a *C*-(3-methylbut-2-en-1-yl) group, which rearranged to a *C*-(2-methylbut-1-en-3-ol) in the presence of TPP and triphenylphosphine, with consequent acid-mediated deprotection to reveal **110**.

**Scheme 19:**
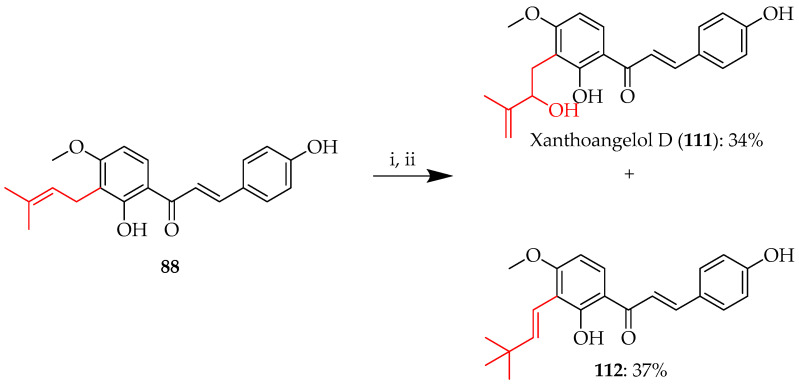
Schenck ene reaction of **88**. Reagents and conditions: (i) *meso*-TPP, O_2_, *hv* (500W), DCM, 5 °C, and 12 h; (ii) Ph_3_P, RT, and 3 h.

Analogously, TPP was employed in a Schenck ene reaction on 4-hydroxyderricin **88** to produce xanthoangelol D (**111**). In addition to the *C*-(3-methylbut-2-en-1-yl) to *C*-(2-methylbut-1-en-3-ol) rearrangement to afford **111**, the isomer **112**, in which the *C*-(3-methylbut-2-en-1-yl) was rearranged to a *C*-(2-methylpent-3-en-2-ol), was also isolated (Scheme 19) [74].

**Scheme 20:**
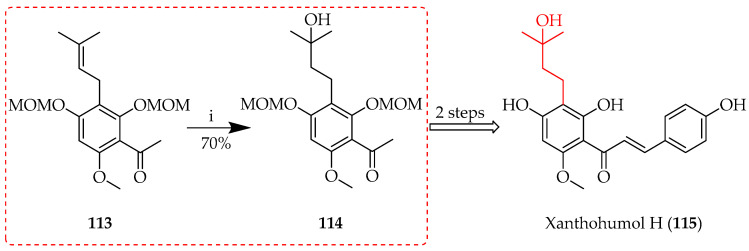
Oxymercuration of **113**. Reagents and conditions: (i) Hg(OAc)_2_-H_2_O, THF, NaBH_4_, NaOH, 30 °C, and 1 h.

Another *C*-prenyl oxidation to transform alkenes into alcohols without concomitant migration is oxymercuration, which uses Markovnikov’s rule in sequential oxymercuration and demercuration [76]. The alkene initially forms a three-membered mercurinium ion intermediate upon interaction with the mercuric acetate (Hg(OAc)_2_) in the presence of water. The more substituted carbon is then attacked by water, forming an intermediate organomercury alcohol. Sodium borohydride (NaBH_4_) mediates demercuration in the second step by substituting a hydrogen atom for the mercury group, producing an alcohol upon insertion of the hydroxyl group on the more substituted carbon [77]. This approach is useful in converting alkenes to alcohols since it is regioselective and does not require carbocation rearrangements. The reaction was used in the total synthesis of xanthohumol H (**115**) [78] to oxidise the prenyl of ketone precursor **113** prior to the Claisen condensation of **114** (Scheme 20).

### 5.3. Cyclisation of Prenyl Functionalities: Cyclic Derivatives

#### 5.3.1. Chromano- and Chromenochalcones

Blending the bioactive characteristics of chalcones with the structural complexity of chromane and chromene systems, chalcones with a prenyl moiety integrated into chromane or chromene structures (X and XII, Figure 1) constitute an intriguing category of compounds. The synergy between the prenylated chromane or chromene moiety and the chalcone backbone may be responsible for the improved biological activities often associated with these derivatives, which includes antioxidant, anti-inflammatory, and anti-cancer properties [79]. Furthermore, the chromane and chromene frameworks’ combination of rigidity and flexibility enhances molecular stability and diversifies potential biological interactions. As a result, these hybrid compounds have generated a great deal of interest and are important as leads for the creation of new therapeutics.

##### *O*-Prenyl Cyclisation

**Scheme 21:**
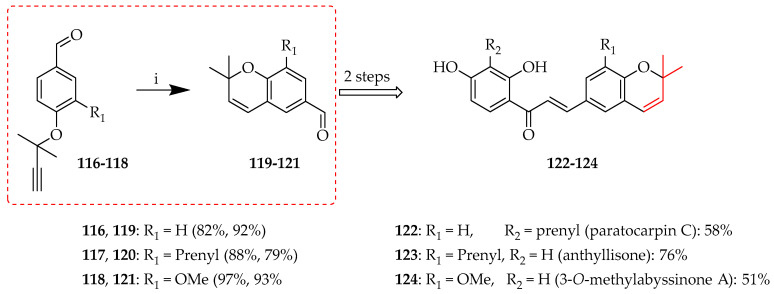
*O*-prenyl cyclisation of **116**–**118**. Reagents and conditions: (i) *N*,*N*-Diethylaniline, sealed tube, 210 °C, and 1.5 h.

*O*-prenyl cyclisation is a chemical process in which a prenyl group attached to an oxygen atom participates in a cyclisation reaction to form a ring structure, typically either a chromane or a chromene system. This reaction is important in the biosynthesis of many natural compounds, including terpenoids and flavonoids, as it results in the creation of complex cyclic ethers or heterocycles. The method typically involves nucleophilic attack of the oxygen atom lone pair on the prenyl group with ensuing ring closure. Synthesis of **119**–**121**, the aldehyde precursors for paratocarpin C, anthyllisone, and 3-*O*-methylabyssinone A (**116**–**118**), respectively [57], was achieved through the cyclisation of **116**–**118** to chromenes **122**–**124** in 82–97% yields using thermal conditions in diethylaniline (Scheme 21).

The choice of reaction conditions can be critical in these cyclisations. While use of Montmorillonite K10 in the presence of DCM on the *O*-prenyl synthon **90** did not lead to cyclisation to chroman **125**, the reaction did occur in the presence of Florisil^®^ and toluene, albeit to a low yield (Table 10) [80].

**Scheme 22:**
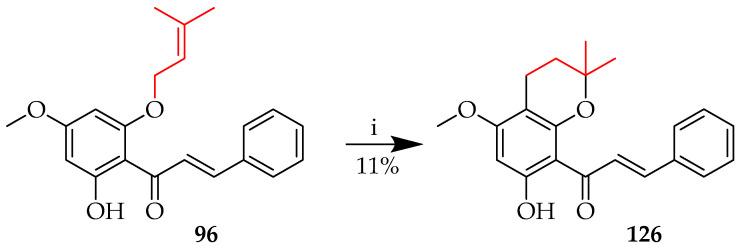
*O*-prenyl cyclisation of **96**. Reagents and conditions: (i) ZnCl_2_, toluene, reflux, and 24 h.

*O*-prenyl cyclisation to a chroman system was also noted among the reaction products of **96** upon treatment with ZnCl_2_ in toluene (see also Section 5.2.2.), whereby synthon **96** gave **126** in an 11% yield. (Scheme 22) [66].

##### *C*-Prenyl Cyclization

**Scheme 23:**
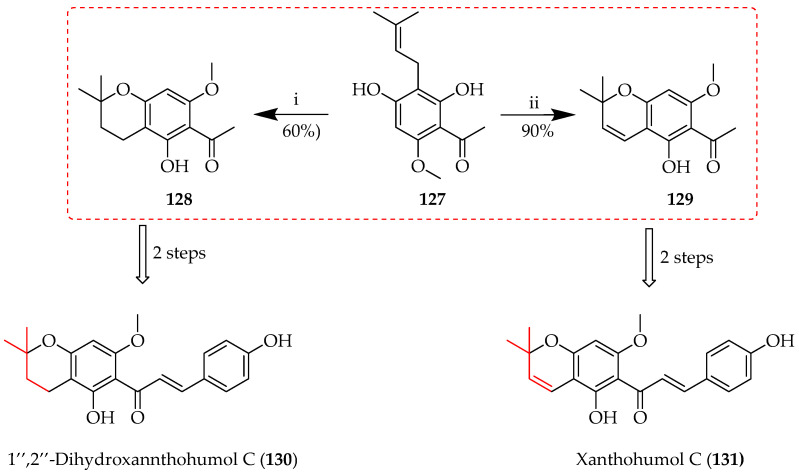
Control of ring oxidation state upon *C*-prenyl cyclization of **127** through control of reaction conditions. Reagents and conditions: (i) Formic acid, THF, reflux, and 3 h; (ii) benzene, dioxane, DDQ, reflux, 3 h.

*C*-Prenyl cyclization involves the attachment of a prenyl group to a carbon atom with subsequent intramolecular cyclization. The reaction typically involves carbocationic intermediates and is essential in the biosynthesis of natural products. Enzymes such as prenyltransferases (see Section 4) assist these processes in nature, while *C*-prenyl cyclisations also feature in synthetic applications, such as Friedel–Crafts alkylation (Section 5.2.1.), to enable the generation of cyclic structures. The syntheses of 1″,2″-dihydroxanthohumol C (**130**) and xanthohumol C (**131**) were achieved via cyclisation of the *C*-prenyl sequence **127** to produce chroman **128** using formic acid in THF and chromene **129** in the presence of 2,3-dichloro-5,6-dicyano-1,4-benzoquinone (DDQ) in dry benzene and dioxane, respectively (Scheme 23) [78].

**Scheme 24:**
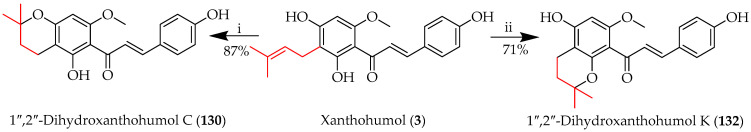
Regioselectivity in *C*-prenyl cyclisation of **3**. Reagents and conditions: (i) TFA, DCM, RT, 5 h; (ii) AlCl_3_, DCM, RT, and 5 h.

Xanthohumol (**3**) can be cyclised to afford either 1″,2″-dihydroxanthohumol C (**130**) or 1″,2″-dihydroxanthohumol K (**132**), depending on the reaction conditions (Scheme 24) [81]. Selective cyclisation to **130** was achieved through reaction with the 4-OH using trifluoroacetic acid (TFA) in DCM, while **3** cyclised to isomeric **132** through reaction with the 2-OH in the presence of AlCl_3_ in DCM.

**Scheme 25:**
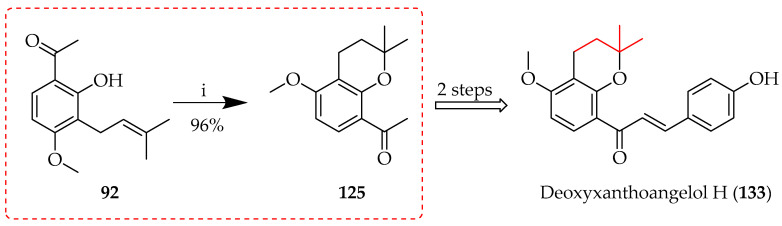
Montmorillonite K10-catalysed cyclisation of **92**. Reagents and conditions: (i) Montmorillonite K10, toluene, 50 °C, and 40 h.

Cyclisation of the *O*-prenyl synthon **90** to **125** using Montmorillonite K10 was unsuccessful, as already mentioned (Table 10); however, when the clay was used for the reaction of *C*-prenyl **92** in toluene under reflux, cyclisation of the prenyl sequence to chroman **125** was successful, affording **125** in a 96% yield (Scheme 25) [80].Intermediate **125** was subsequently used as the ketone precursor for deoxyxanthoangelol H (**133**).

**Scheme 26:**
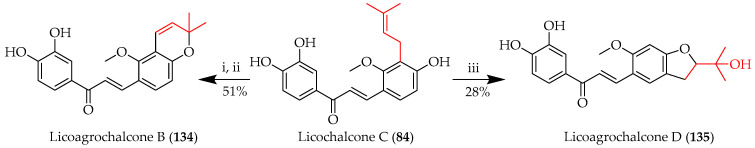
*C*-prenyl cyclisation of **84**. Reagents and conditions: (i) PhSeCl, DCM, −30 °C, and 15min; (ii) H_2_O_2_, THF, RT, and 12 h; and (iii) *m*-CPBA, DCM, 0 °C-RT, and 12 h.

Licoagrochalcone B (**134**), another prenylated chalcone with a chromene system, was synthesised from licochalcone C (**84**) by the cyclisation of the *C*3-prenyl through reaction with the 4-OH in the presence of phenylselenyl chloride, followed by treatment with aqueous H_2_O_2_ in THF. On other hand, Licoagrochalcone D (**135**), featuring a five-membered indan system, was also obtained from **84** upon treatment with *meta*-chloroperoxybenzoic acid (*m*-CPBA) (Scheme 26) [82].

**Scheme 27:**
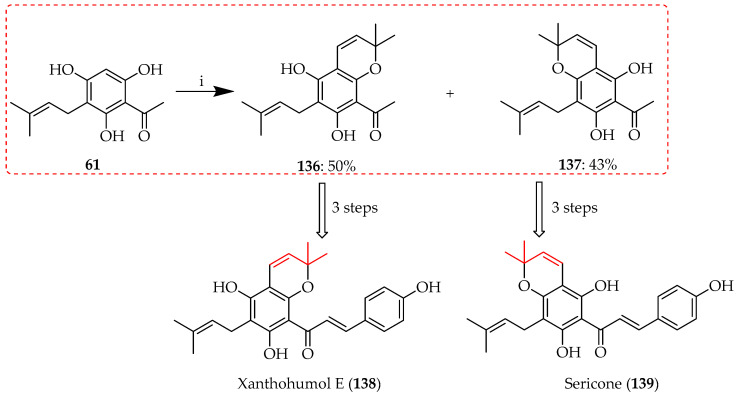
Direct cyclisation of **61** to chromenes **136** and **137**. Reagents and conditions: (i) 3-Methyl-2-butenal, EDDA, DCM, RT, and 12 h.

Direct cyclisation upon integration of a prenyl group is illustrated by the use of ethylenediamine diacetate (EDDA) in DCM in the presence of the prenylation agent, 3-methyl-2-butenal. The cyclisation of **61** took place with either available hydroxy group, at either the 4-OH or 6-OH positions, to produce two chromene products, **136** and **137** (Scheme 27) [58]. Chromenes **136** and **137** proved to be useful intermediates in the synthesis of the chalcones xanthohumol E (**138**) and sericone (**139**), respectively.

**Scheme 28:**
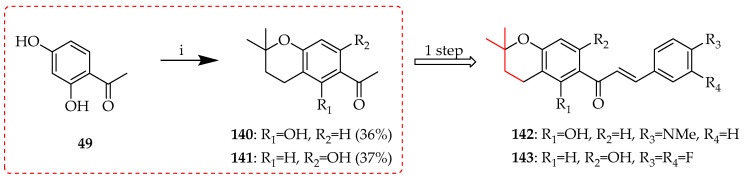
Direct cyclisation of **49** to chromans **140** and **141**. Reagents and conditions: (i) isoprene, BF_3_·Et_2_O, 1,4-dioxan, RT, and 12 h.

The use of isoprene as a prenylation agent for **49** in the presence of BF_3_·Et_2_O in 1,4-dioxan afforded the acetylated chromans **140** and **141** through cyclisation with either C2 or C4 hydroxy groups, respectively; their coupling with appropriate aldehydes then afforded chromanochalcones **142**–**143** (Scheme 28) [63].

**Scheme 29:**
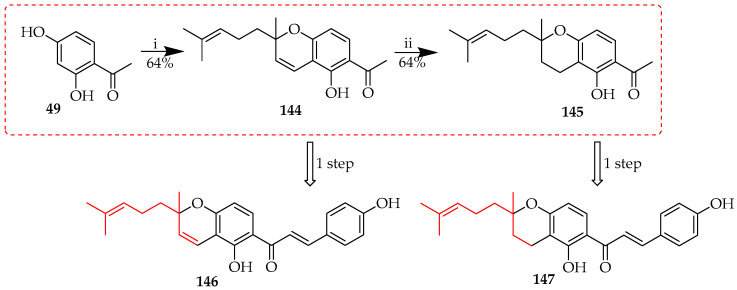
Direct cyclisation of **49** to chromene **144** followed by reduction to **145**. Reagents and conditions: (i) citraldimethylacetal, pyridine, 150 °C, and 11 h; and (ii) Pd/C, methanol, RT, and 3 h.

As seen in Scheme 20, Scheme 21, Scheme 22, Scheme 23, Scheme 24, Scheme 25, Scheme 26, Scheme 27 and Scheme 28, *O*-prenyl cyclisations typically result in 2,2-dimethyl substituted chromano- and chromenochalcones. Similar ring systems, but with a 2-methyl-2-(4-methylpent-3-en-1-yl) substitution at C-2, may be prepared through the intramolecular reaction of monoterpenoid (C10 or geranyl) fragments. This is illustrated by Scheme 29, where **144** was prepared through the initial pyridine-catalysed condensation of compound **49** with citraldimethylacetal, yielding acetylchromene **144**. This intermediate was then reduced to acetylchroman **145**. Both **144** and **145** underwent subsequent Claisen–Schmidt condensation with aromatic aldehydes to produce the target chalcones **146** and **147** [63].

Overall, the diverse synthetic strategies for prenylated chalcones, ranging from simple mono-prenylation reactions to more advanced sigmatropic rearrangements and prenyl cyclisations, highlight the versatility and adaptability of prenylated chalcone synthesis. The careful choice of reaction conditions is critical for the reaction outcome and yield. These methodologies not only offer efficient routes for generating a wide variety of prenylated chalcone derivatives but also enable structural modifications crucial for enhancing biological properties for exploring the pharmacological potential of prenylated chalcones. The following section will explore the biological activities of these compounds, emphasising the relationships between structural features and bioactivity.

## 6. Biological Activities of Prenylated Chalcones

Prenylated chalcones display a wide range of biological activities, often enhanced by the physicochemical properties of the lipophilic prenyl groups, as mentioned previously. In this section we describe these activities, with a focus on the literature over the past 10 years.

### 6.1. Anti-Cancer Potential of Prenylated Chalcones

Cancer is characterised by the uncontrolled proliferation of abnormal cells, which have the ability to spread to surrounding tissues. Metastasis is the process by which this growth spreads to distal areas of the body. Environmental effects, lifestyle choices, and genetic mutations are among the risk factors for carcinogenesis. Depending on the type and stage of cancer, treatment may include radiation, chemotherapy, surgery, or targeted therapies, with several existing chemotherapies derived from natural products [83].

Prenylated chalcones have been found to possess a broad spectrum of anti-cancer activity on a variety of cancer cell lines, mainly by inducing apoptosis (programmed cell death), cell cycle arrest, or inhibiting metastasis. They may disrupt several signalling pathways that contribute to tumour growth, including the phosphoinositide 3-kinase/protein kinase B (PI3K)/Akt and nuclear factor-kappa B (NF-κB) pathways, which are both essential for the survival and growth of cancer cells [84]. Furthermore, because prenylated chalcones have anti-angiogenic properties, they can prevent the development of new blood vessels [85], which are necessary for tumour growth and nutrition. Prenylated chalcones are interesting prospects for creating novel anti-cancer treatments because of these multidimensional effects, and they may have less adverse effects than traditional chemotherapy. Understanding these compounds’ exact mechanisms of action and improving their structures for increased safety, efficacy, and bioavailability in the treatment of cancer are the main goals of the current research.

#### 6.1.1. Anti-Cancer Effects of Naturally Occurring Prenylated Chalcones

Licochalcone A (**2**) is recognised for its potent anti-cancer properties across diverse cell types. It (**2**) exerts its effects through multiple mechanisms, including inducing apoptosis, regulating the cell cycle to inhibit proliferation, suppressing invasion and metastasis, and inhibiting angiogenesis. It (**2**) was shown to exert a cytostatic effect on HONE-1, NPC-39, and NPC-BM cells, promoting apoptosis via the mitochondrial pathway and the JNK/p38 signalling cascade at concentrations of 20–80 µM [86]. Various mechanisms were postulated in the breast cancer cell line MCF-7, including the activation of LC3-II signalling, endogenous pathway-mediated apoptosis, and mitochondrial dysfunction, at concentrations between 10 and 50 µM [87,88,89]. It (**2**) also demonstrated strong activity on glioma cell lines, including U87, M059K, U-251, GBM8901, and GSC, at 5 to 40 µM. Mechanistically, **2** inhibited cell growth by inducing cell cycle arrest in the G0/G1 and G2/M phases, mitochondrial fragmentation and ADAM9 expression, and, at low concentrations (<12.5 µM), specifically induced cell death in glioma stem cells (GSCs), with almost all GSCs dying after 6 days of treatment, while both normal fibroblasts and neural stem cells were unaffected [90,91,92]. Compound **2** demonstrated significant anti-lung cancer effects across various cell lines. It reversed lung injury induced by the tobacco carcinogen 4-(methyl-nitrosamino)-1-(3-pyridyl)-1-butanone (NNK) via modulation of the miR-144 and mitogen-activated protein kinase (MAPK) pathways at 10 µM, suggesting potential applications in both lung cancer and lung injury [93]. It (**2**) induced UPR, autophagy, and apoptosis in H292 lung cancer cells by upregulating miR-144-3p. At 10 μM, **2** increased miR-144-3p, which downregulated Nrf2 and promoted ER stress, enhancing apoptosis and growth inhibition. At 40 μM, **2** elevated the C/EBP homologous protein (CHOP) protein but failed to activate its pro-apoptotic targets, suggesting the inhibition of CHOP-dependent apoptosis [94]. In A549 and H460 cells, **2** (20, 40, or 60 μM) blocked cell cycle progression at the G2/M phase and induced apoptosis [95]. Similarly, in A549 and H1299 cells, **2** activated the CHOP pathway, contributing to its pro-apoptotic effects while not affecting human embryonic lung fibroblasts [96]. It (**2**) was investigated for its effects on malignant pleural mesothelioma, where it inhibited cell growth in MSTO-211H and H28 cells, with IC_50_ values of ~26 and 30 µM, respectively, and suppressed Sp1 expression along with the downstream targets cyclin D1, Mcl-1, and survivin. Mechanistically, **2** activated the mitochondrial apoptotic pathway by altering the Bax/Bcl-xL ratio, inducing Bid-mediated mitochondrial membrane loss, caspase activation, and nuclear fragmentation. Flow cytometry confirmed annexin V/PI-positive apoptotic cells. These findings suggested that **2** induced apoptosis in MPM cells via Sp1 downregulation and mitochondrial pathway activation [97]. In hepatoma (HepG2) cells, with IC_50_ values of 65.96 μM (24 h) and 44.13 μM (48 h), **2** induced morphological changes, reactive oxygen species (ROS) generation, G2/M arrest, and apoptosis. It downregulated cell cycle genes (survivin, cyclin B1, and *CDK1*) while upregulating Wee1, P21, cyclin D1, and JNK1 [98,99]. In HuH7 and HepG2 lines, **2** triggered autophagy through ULK1/Atg13 activation and ROS accumulation [100]. Gastric cancer cells (MKN45, SGC7901, and GES-1) underwent growth inhibition via blockade of the Akt/HK2 axis [101], while in BGC-823 cells, it paradoxically activated extracellular signal-regulated kinase (ERK), c-Jun N-terminal kinase (JNK) [102]. In squamous carcinoma (FaDu) cells, there was upregulation of the tumour necrosis factor-related apoptosis-inducing ligand (TRAIL) through ERK [103]. Compound **2** inhibited proliferation and induced apoptosis in T24 bladder cancer cells through mitochondrial and ER stress-related pathways. In the presence of **2**, intracellular ROS and Ca^2+^ levels increased, while the mitochondrial membrane potential was lowered, and Apaf-1, caspase-9, and caspase-3 were upregulated, indicating mitochondrial dysfunction. Calpain 2 and caspase-4 were also activated, linking apoptosis to ER stress. These results suggest that **2** triggers T24 cell death via combined mitochondrial and ER stress-mediated mechanisms [104].

Recently, **2** exhibited anti-cancer activity against HCT-116, resulting in G0/G1 phase arrest, apoptosis, and high ROS generation, which were attenuated by the ROS inhibitor *N*-acetyl-L-cysteine. The targeting of thioredoxin reductase 1 (TrxR1) in HCT-116 cells led to high ROS levels and apoptosis [105]. Substrate **2** potently suppressed hypoxia-induced factor (HIF)-1α accumulation and the expression of HIF-1α target genes, including GLUT1 and PDK1 in HCT116 cells, and effectively inhibited ATP production [106]. Substrate **2** inhibited non-small cell lung cancer (NSCLC) cell growth and induced apoptosis by destabilising proteins including survivin, XIAP, and RIP1 without altering their mRNA levels. It also activated ERK and p38 while suppressing JNK, leading to cytoprotective autophagy [107].

It (**2**) markedly decreased the viability and caused apoptosis in several endometrial cancer (EMC) cell lines (Hec1A (HTB-112), AN3CA (HTB-111), HEC59 (JCRB1120), and Ishikawa (JCRB1505)) and primary (EMC-7) cells. Additionally, it was discovered that **2** causes endoplasmic reticulum (ER) stress, which causes EMC lines to express more ER-related proteins (GRP78/PERK/IRE1α/CHOP). In human EMC cells treated with **2**, suppression of GRP78 expression dramatically diminished the effects of **2**, leading to the decreased production of proteins associated with ER and apoptosis, as well as ER stress-mediated cell death [108]. Compound **2** also suppressed glioma growth, migration, and invasion by inducing mitochondrial dysfunction and ROS production, while ROS inhibition reversed these effects. In vivo, treatment with **2** suppressed glioma growth in nude mice in a dose-dependent manner: at 10 μM, the tumour volume and weight were reduced by about half compared to vehicle control, and at 20 μM, they were reduced by ~80%. Imaging and immunohistochemistry confirmed that **2** inhibited proliferation (Ki-67), reduced angiogenesis (CD-34), and promoted apoptosis (TUNEL), suggesting its anti-glioma activity involves apoptosis through ATM/ATR pathway activation and oxidative stress [109]. In a 28-day study of uterine leiomyoma, BALB/c nude mice bearing ELT3 tumours were treated with **2** (10 or 20 mg/kg, orally). Treatment significantly suppressed tumour growth and weight in a dose-dependent manner without affecting body weight or causing toxicity to major organs. Histology and immunohistochemistry showed reduced Ki67 expression, while molecular analysis revealed the upregulation of p-JNK, p-NRF2, ER stress proteins (GRP78/CHOP), and apoptotic protein c-caspase-3. It activated the GRP78/IRE1α/ATF6/CHOP pathway, upregulated caspase-dependent proteins, and its effects were reversed by NAC or Z-VAD-FMK. Blood markers (GOT, GPT, BUN, and creatinine) remained unchanged, confirming safety. These results highlighted the potential as a therapeutic agent via the JNK/GRP78/NRF2 signalling axis [110].

Various studies have explored the anti-tumour effects of xanthohumol (**3**) against glioma. It (**3**) upregulated miR-4725-3p in U87-MG and Hs-683 cells, leading to the downregulation of Stim1, a calcium sensor involved in invasion [111]. In U87 cells, **3** reduced viability and induced apoptosis through caspase activation, mitochondrial dysfunction, ROS generation, and Bcl-2 family regulation. It (**3**) upregulated miR-204-3p via the ERK/c-Fos pathway, and miR-204-3p overexpression enhanced glioma apoptosis. MiR-204-3p directly targets IGFBP2, inhibiting the IGFBP2/AKT/Bcl-2 pathway [112]. In colorectal cancer cells, mechanistic experiments demonstrated that **3** triggered DNA damage, inducing the phosphorylation of ATM/ATR, γ-H2AX accumulation, and p53/p21 activation, leading to the S phase or G1 arrest. Synergistic potential may be indicated by the observation that sub-cytotoxic concentrations of **3** (2.5–10 µM) sensitised resistant SW480 cells to the active metabolite of irinotecan (SN38). The prenyl group of **3** was associated with the facilitation of nuclear uptake and DDR activation, allowing it to act as both a direct cytotoxic and chemosensitising adjuvant [113]. In melanoma, **3** showed dose-dependent cytotoxicity and inhibited melanoma growth and metastasis. It reduced the proliferation, colony formation, and migration of Mel Ju and Mel Im cells at subtoxic concentrations (≤30 µM) but spared hepatocytes at up to 100 µM. In a murine model of hepatic metastasis, the continuous delivery of **3** at 10 mg/kg/day reduced hepatic B16-F10 metastases, with a lower Ki67 expression and greater necrosis within lesions. Again, lipophilicity and cell uptake was associated with prenylation. This observed selectivity of **3** in impairing proliferation and migration in melanoma cells led to **3** being touted as a promising candidate for targeting melanoma progression and liver metastasis [114]. In canine haematological models, **3** and seven derivatives (natural and semisynthetic) were active against three cancer cell lines: CLBL-1 (B-cell lymphoma), CLB70 (B-cell leukaemia), and GL-1 (B-cell leukaemia). All compounds showed dose-dependent cytotoxicity (0.1–30 µM, 48 h), but the most potent were **3** (IC_50_ = 1.3–6.3 µM), xanthohumol D (**148,** IC_50_ = 0.55–5.3 µM), and the 4′-*O*-β-D-(4′′′-*O*-methyl)-glucopyranoside of **3** (**149**, IC_50_ = 1.2–4.9 µM). All three compounds induced apoptosis, decreased mitochondrial potential, and increased ROS production (except in resistant GL-1 cells). Western blotting confirmed the downregulation of anti-apoptotic Bcl-2, particularly in CLBL-1 and CLB70. SAR analysis defined both the chalcone skeleton and uncyclised prenyl groups of **3**, **148,** and **149** as crucial for potency, that cyclisation of the prenyl unit reduced activity, and that glycosylation preserved cytotoxicity, while potentially improving solubility and bioavailability [115].


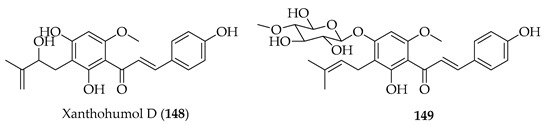


The cytotoxicity of **3** was also evaluated in human hepatocellular carcinoma (HepG2) cells. It (**3**) inhibited HepG2 proliferation in a dose- and time-dependent manner, with IC_50_ values of 68 µM (24 h) and 52 µM (48 h). Apoptosis assays showed that **3**, at 25 µM, induced apoptosis in ~47% of HepG2 cells, increasing to >80% at 100 µM, mediated by caspase-3 activation and PARP cleavage. In contrast, primary rat hepatocytes were highly resistant: even at 100 µM **3**, viability was >90% with no apoptosis. The chalcone scaffold and prenyl side chain were considered to enable selective apoptosis, likely through preferential uptake and mitochondrial disruption in HepG2 cells [116].

In NSCLC cells, **3** was demonstrated to directly inhibit T-lymphokine-activated killer cell-originated protein kinase (TOPK), a serine/threonine kinase implicated in tumour proliferation and metastasis. At 20 µM, **3** reduced the viability of A549 and HCC827 cells by >80% after 72 h but not normal lung (MRC-5) fibroblasts. It induced G0/G1 cell cycle arrest, enhanced apoptosis, and suppressed migration and invasion. In an A549 xenograft model, a daily i.p. injection of **3** at 25 mg/kg reduced the tumour weight by 50% without causing hepatotoxicity or nephrotoxicity. Mechanistically, **3** was demonstrated to directly bind the ATP-binding pocket of TOPK (KD = 2.9 µM), inhibiting phosphorylation of histone H3 and Akt, both in vitro and in xenografts. The prenyl chain at C-3′ and the methoxy group at C-6′ were crucial for potency, with **3** (IC_50_ = 11.6 µM) more active than xanthohumol D (**148**) (28 µM) and desmethylxanthohumol (**62**) (55 µM), correlating with stronger TOPK inhibition. Prenylation and methoxylation thus conferred superior TOPK binding and anti-cancer activity and supported **3** as a promising TOPK inhibitor [117].

An optimised solid lipid nanoparticle formulation of **3** has been investigated to overcome its limited oral bioavailability and sub-optimal pharmacokinetics. Compared to **3**, the formulation showed a 4.7-fold increase in systemic exposure, a 6.5-fold longer half-life, and nearly 4800% relative bioavailability in vivo. In PC-3 prostate cancer cells, encapsulated **3** produced sustained cytotoxicity, with enhanced inhibition compared to **3** and with effects comparable to the control, camptothecin. The results highlighted that while the prenyl side chain of **3** confers lipophilicity, it also limits solubility, and that formulation choice can overcome absorption barriers, enabling improved delivery to target cells and facilitating translational studies [118].

Isobavachalcone (**80**) was identified as a potent and non-toxic inducer of autophagic flux in human and murine cells. It (**80**) inhibited AKT phosphorylation and downstream mTORC1 activity, leading to activation of the pro-autophagic transcription factors TFEB and TFE3. Also noted were the induction of ER stress (PERK/eIF2α phosphorylation, CHOP and ATF6 activation, and XBP1 splicing), showing crosstalk between the unfolded protein response and autophagy. In vivo, **80** injections reduced AKT/mTOR/S6K signalling and increased LC3-II, a marker of autophagy. It (**80**) also enhanced immunogenic cell death (ICD): in combination with low-dose mitoxantrone or oxaliplatin, **80** increased the ATP release from cells, promoted dendritic/T-cell infiltration, decreased Tregs, increased the CD8^+^/Treg ratio, and lowered the PD-1 expression on cytotoxic T-lymphocytes. These effects were lost in the presence of constitutively active AKT, Atg5 knockout, or PERK deficiency, confirming mechanistic dependence. Again, the prenylation of **80** and its impact on lipophilicity was held to enable the dual targeting of AKT/mTOR and ER stress pathways. Substrate **79** alone had little cytotoxicity but showed synergy with ICD-inducing chemotherapy, boosting anti-cancer immune responses [119].

Substrate **80** was investigated for its dual role in modulating multidrug resistance (MDR). Substrate **80** was largely non-toxic to HT29 colorectal adenocarcinoma and the doxorubicin-resistant variant HT29/Dx up to 40 µM, though it showed moderate cytotoxicity to kidney (MDCK) cells (IC_50_~26.6 µM). As **80** stimulated the growth of ABCB1-overexpressing MDCK-MDR1 cells at 10–20 µM, an effect abolished by verapamil, **80** was considered both a substrate and competitive inhibitor of ABCB1. Fluorescence assays confirmed that **80** increased doxorubicin and rhodamine 123 accumulation in resistant cells but did not fully reverse resistance. Differential scanning calorimetry showed that **80** intercalated into phosphatidylcholine bilayers, lowering transition temperatures and enthalpy, broadening phase transitions, and identifying it as an effective membrane perturbing agent. Molecular modelling indicated amphiphilicity (logP = 4.19) and favourable bilayer partitioning (ΔG = −4.81 kcal/mol), supporting both transporter binding and lipid interactions. Prenylation at C-6 was noted to affect the amphiphilic balance, enabling **80** to act on both membrane bilayers and ABCB1 transporters. Overall, **80** was considered to interfere with MDR through both membrane-disruptive and transporter-inhibitory mechanisms [120].

Substrate **80** has also been evaluated against the CNS cancer, glioblastoma (GBM), using cell and xenograft models. Substrate **80** inhibited the proliferation, migration, and invasion of U87MG (p53 wild-type) and U251 (p53 mutant) cells, with IC_50_ values of 4.4 and 1.9 µM at 48 h, respectively, compared to the reference therapy temozolomide (224–473 µM). In subcutaneous and orthotopic GBM xenografts, daily **80** (20–40mg/kg p.o.) suppressed tumour growth without causing systemic toxicity, decreased angiogenesis (CD31 ↓), and increased apoptosis (cleaved caspase-3 ↑). Substrate **80** was shown to bind directly to ERα (ESR1), to suppress NLRP3 inflammasome transcription, alleviate pyroptosis (NLRP3/ASC/GSDMD/IL-1β/IL-18 ↓), and promote mitochondria-dependent apoptosis via Bcl-2/Bax modulation and caspase-3 activation. Prenylation was associated with blood–brain barrier (BBB) permeability and engagement with ERα and to underpin both anti-inflammatory (pyroptosis inhibition) and pro-apoptotic activity. Prenylation of **80** was considered to optimise the compound for CNS druggability and to facilitate the dual modulation of pyroptosis/apoptosis in GBM [121]. Both **80** and bavachalcone (**83**) were also evaluated using the human leukaemia K562 cell line. SAR analysis indicated that prenylation at the 5 position markedly improved their cytotoxic effects. Substrate **83** exhibited stronger activity, with an IC_50_ of 2.7 μM. Morphological observations and annexin V/PI staining revealed that **80** and **83** inhibited K562 cell proliferation, primarily by inducing apoptosis [61].

Paratocarpin E (**108**) has anti-cancer potential against various cancer cell lines, including breast, leukaemia, and kidney cancer cells. Substrate **108** exhibited significant cytotoxicity, notably against MCF-7 breast cancer cells, with an IC_50_ of 19.6 μM. Treatment with **108** induced classical apoptotic features in MCF-7 cells by increasing the activation of caspase-8, caspase-9, and inducing PARP cleavage. Mechanistically, it induced apoptosis by activating the p38 and JNK MAPK signalling pathways and suppressing ERK signalling [122].

Isocordoin (**150**) has been evaluated for cytotoxic activity against colorectal (HT-29), breast (MCF-7), and prostate (PC-3) cancer cells, along with non-malignant colon cells (CCD841). Substrate **150** had the greatest toxicity against PC-3 cells (IC_50_ = 15.2 µM, SI = 5.2), followed by MCF-7 (21.1 µM, SI = 3.7) and HT-29 (27.2 µM, SI = 2.9). Unlike daunorubicin, which lacked selectivity, **150** displayed moderate potency with improved safety. Mechanistic studies in HT-29 and MCF-7 cells revealed mitochondrial dysfunction (loss of ΔΨ m), ROS modulation, and caspase-3 activation, consistent with the induction of apoptosis. Docking studies showed that **150** bound to caspase-3 (ΔG = −6.13 kcal/mol, Ki = 32 µM), supported by H-bonding (Trp214, Phe250) and π-interactions (Phe256, Trp206, and Phe247). SAR analysis attributed the increased lipophilicity and caspase-3 binding affinity to the isoprenyl side chain, making **150** more active than its non-prenylated analogue (2′,4′-dihydroxychalcone, IC_50_ > 100 µM). The α,β-unsaturated carbonyl and hydroxylation pattern were considered hallmarks of apoptotic activity. In silico pharmacokinetic predictions of **150** were favourable. Overall, **150** was held as a promising prenylated chalcone with selective cytotoxicity against prostate and breast cancer, mediated by caspase-dependent apoptosis and enhanced by prenylation-driven lipophilicity [123].


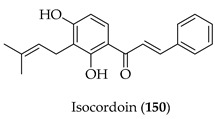


Kanzonol C (**151**) and the related licoagrochalcone A 4′-methylether (**11**) were evaluated for antiproliferative activity, with **151** more active against MDA-MB468 triple-negative breast cancer cells (IC_50_ = 5.97 µM) and MCF-7 breast cancer cells (IC_50_ = 10.10 µM), exceeding that of cisplatin in MCF-7 cells (cisplatin IC_50_ = 21.54 µM). Synthetic derivatives of **151**, namely hexahydro-kanzonol (**152**) and triacetyl-kanzonol (**153**), were less active with IC_50_s > 25 µM, emphasising the importance of unsaturation within the prenyl groups and unmodified phenolic hydroxy groups for activity. Isobavachalcone (**80**) and **11** were also weaker (IC_50_s > 25–50 µM). As **151** was non-toxic to normal liver HepaRG cells up to 50 µM, this suggested a good safety margin [36].


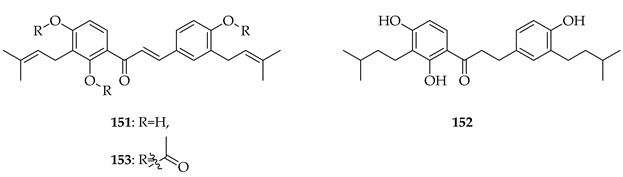


#### 6.1.2. Anti-Cancer Effects of Synthesised Prenylated Chalcones

In addition to the native natural products discussed in Section 5.1.1, many derivatives of these lead compounds have been synthesised and evaluated for anti-cancer activity. Imidazole-modified chalcones based on **2** were designed to improve potency and selectivity against prostate cancer cells. Substrate **2** itself inhibited proliferation across both androgen receptor (AR)-positive (LNCaP, 22Rv1) and AR-negative (PC-3, DU145) cell lines, with IC_50_ values of 15.7–23.3 µM. Among the prepared derivatives, compounds **154**, **155**, **156**, and **157** retained their potency, especially in AR-positive lines, suggesting an increased selectivity toward AR signalling-dependent cells. Imidazole-modified chalcones **158** and **159** were the most potent, with IC_50_ = 9.4–9.8 µM in LNCaP and ~28 µM in 22Rv1 compared to the standard enzalutamide (IC_50_ = 21.7 µM in LNCaP, 67.5 µM in 22Rv1). Bulky isobutyl or pentyl groups on the imidazole moiety led to enhanced AR-positive selectivity, while shorter alkyl chains reduced activity. Key concepts from the study were that the prenyl group was crucial for lipophilicity and uptake, that imidazole substitution enhanced AR-positive selectivity, and that bulky alkyl substitutions favoured potency against LNCaP/22Rv1 but not AR-null PC-3/DU145. The imidazole-modified analogues of **2** were considered to be selective anti-prostate cancer agents with potential advantages over existing therapies [124].


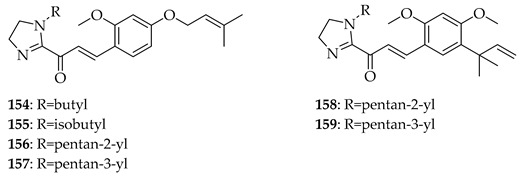


A comparison of **3** with its semisynthetic aurone derivative (*Z*)-6,4′-dihydroxy-4-methoxy-7-prenylaurone (**160**) was undertaken using ten human cancer cell lines (breast, colon, prostate, lung, and leukaemia) and two normal lines (human endothelial HLMEC, murine fibroblasts BALB/3T3). Both **3** and **160** exhibited potent-to-moderate antiproliferative activity (IC_50_ ≈ 7–20 µM in breast cancers, leukaemia, and doxorubicin-resistant colon LoVo/Dx; weaker in HT-29 colon cells). Aurone **160** showed a higher selectivity than **3**, with SI values up to 7.09 in LoVo/Dx and ~5.5 in breast cancer lines, while cisplatin displayed poor selectivity. Mechanistic interpretation highlighted that prenylation drives potency, while the aurone led to increased selectivity and reduced cytotoxicity 1.5–2.3-fold toward normal cells. Also, unlike **3**, **160** cannot be metabolised into 8-prenylnaringenin, a potent phytoestrogen that may promote estrogen-dependent cancers, suggesting improved safety. Thus, while the prenyl group is essential for activity, aurone cyclization reduces the off-target estrogenic risk while maintaining antiproliferative potency, making aurone derivatives potentially more selective anti-cancer leads [125].


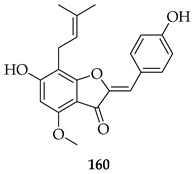


Many analogues of **3** have been synthesised to evaluate their anti-angiogenic activity in vitro. These compounds significantly inhibited several angiogenesis-related functions of human umbilical vein endothelial cells (HUVECs), including proliferation, adhesion, migration, invasion, and tube formation at 10 µM. Notably, **161,** a fluorinated analogue with a *p*-methoxy group on the B-ring, showed the strongest activity [126].


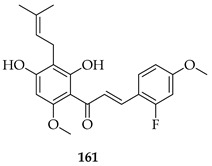


The novel 3′,5′-diprenylated pyridyl chalcone (**162**) demonstrated potent anti-cancer activity against prostate cancer PC3 cells. In vitro, **162** inhibited proliferation, with IC_50_ values of 4.56, 4.67, and 3.55 μM at 24, 48, and 72 h, respectively, showing a stronger selectivity for PC3 over DU145 and normal RWPE-1 cells. Substrate **162** caused cell cycle arrest at the sub-G1 phase (53.5% cells at 8 μM) and triggered programmed cell death via dual pathways: caspase-dependent apoptosis and gasdermin E-mediated pyroptosis. The upregulation of PKCδ and activation of the JNK pathway were observed, leading to Bax upregulation, caspase-3 cleavage, PARP cleavage, IL-6/IL-1β release, and gasdermin E-N formation. PKCδ knockdown or JNK inhibition suppressed both apoptosis and pyroptosis, confirming pathway dependence. In vivo, **162** (30–60 mg/kg i.p.) significantly suppressed PC3 xenograft growth without toxicity, upregulating PKCδ and IL-6 while reducing the proliferating cell nuclear antigen (PCNA) and Bcl-2 expression. The diprenylation pattern of **162** at the 3′ and 5′ positions was associated with enhanced potency, lipophilicity, and pathway activation compared to non-prenylated chalcones, enabling a dual apoptotic/pyroptotic mechanism. Overall, **162** was considered a promising lead compound, exploiting apoptosis–pyroptosis crosstalk to overcome resistance in prostate cancer [127].


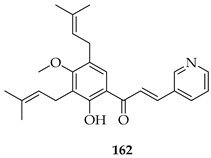


The same group later prepared an additional series of thirty-seven derivatives. Among these, **163**, bearing a diethylaminoethoxy side chain, showed the strongest activity. It inhibited the proliferation of both LNCaP prostate cancer cells (IC_50_ = 2.33 µM) and K562 leukaemia cells (IC_50_ = 2.38 µM) comparably to doxorubicin (1.36–2.17 µM). Also, **163** displayed a slightly better selectivity index (SI) of 1.97 than doxorubicin (SI = 1.74), indicating a relatively lower toxicity toward normal LX-2 liver cells. Mechanistic studies showed that **163** induced apoptosis and necrosis in both cell lines, caused cell cycle arrest (S phase in LNCaP, G2/M in K562), and directly targeted the PI3K/AKT pathway, downregulating pPI3K and pAKT (Ser473) levels. Docking and thermal shift assays confirmed AKT1 as a direct target, suggesting that prenylation coupled with the introduction of a diethylaminoethoxy chain at C-4 optimised the binding affinity and potency [128].


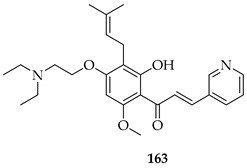


Seventeen isobavachalcone (**80**) derivatives were synthesised and tested for cytotoxicity against three human lung cancer cell lines. Among them, compound **164** showed the strongest activity, particularly against H1975 and A549 cells, with IC_50_ values of 4.35 and 14.21 μM, respectively. Compound **164** induced apoptosis through increasing the Bax/Bcl-2 ratio, elevating cytochrome c, downregulating Akt, and activating caspase-9 and -3. It triggered necroptosis by upregulating receptor-interacting protein kinase 3 (RIP3) and the mixed lineage kinase domain-like protein (MLKL). Additionally, it caused mitochondrial dysfunction, reduced ATP levels, and led to excessive ROS accumulation [129].


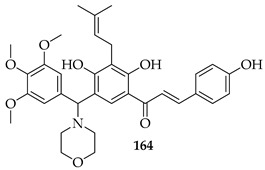


Nine novel prenylated chalcones were synthesised and screened against castration-resistant prostate cancer lines DU145 and PC3. The most active compounds were **165** and **166**. Both displayed potent cytotoxic activity, with IC_50_ values of 54.96 µM (PC3) and 88.73 µM (DU145) for **165** and 57.22 µM (PC3) and 61.71 µM (DU145) for **166**, with favourable selectivity. Mechanistic studies showed that both induced apoptosis via mitochondrial membrane depolarization, phosphatidylserine externalisation, and cytoskeleton disruption. SAR analysis emphasised that halogen substitution (B-ring *meta*-F in **165**, A-ring *ortho*-Cl in **166**), combined with *O*-prenylation, significantly improved potency compared to other analogues, highlighting the impact of both prenylation and halogen positioning. Both **165** and **166** were considered promising prenylated scaffolds against resistant prostate cancers [130].


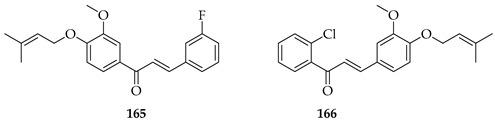


*O*-prenylated chalcone **167** was found to arrest lung cancer (NCI-H460) cells during mitosis, causing spindle collapse and chromosome misalignment, which activated the spindle assembly checkpoint, ultimately leading to cell death [131]. In an attempt to investigate the mechanism underlying the activity of **167**, yeast growth-inhibitory and p53 MDM2 transactivation assays were used to evaluate the compound in HCT116 human colon adenocarcinoma cells. The potency of **167** was ascribed to activation of the p53 pathway, leading to cell cycle arrest and mitochondria-dependent apoptosis [132]. Based on the promising results with **167**, a library of related *O*-and/or *C*-prenylated chalcones was synthesised, alongside the evaluation of their cytotoxic effects on human colon cancer HCT116 cells. Among the compounds, **168**, *O*-prenylated and with a bromo at R_2_, was the most potent, again highlighting the potential positive impact of halogenation and showing parallels with compound **165** [133].


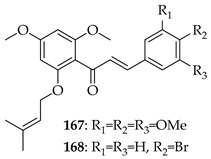


Two synthetic prenylated chalcones, **169** and **170**, were evaluated for their effects on prostate cancer (PC3) cells through the modulation of Bloom (BLM) helicase, a key enzyme in DNA repair and genomic stability. Both compounds suppressed cell proliferation, induced apoptosis, and caused cell cycle arrest at the G2/M phase, but **169** was more potent with an IC_50_ of 0.375 µM, nearly ten times stronger than **170** (IC_50_ = 3.86 µM). Mechanistic assays revealed that both compounds selectively decreased BLM helicase protein expression without altering its mRNA levels, suggesting post-transcriptional regulation at the translation level. In enzymatic assays, **169** and **170** inhibited the ATPase and DNA-unwinding activities of purified BLM helicase while sparing other helicases, demonstrating high target selectivity. In addition, **169** showed stronger inhibition of helicase ATPase activity than **170**, correlating with its superior cytotoxicity. These results were considered to identify compounds with a novel anti-cancer mechanism distinct from traditional apoptotic or ROS-based pathways [134].


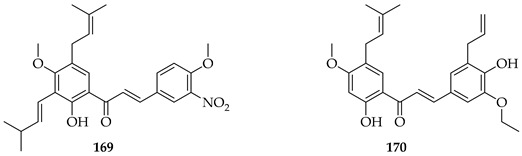


A series of eleven novel *O*-prenylchalcones were tested against gastric cancer (AGS) cells, with most showing a dose-dependent reduction in viability and with IC_50_s ranging from 26 to 60 µM. Among them, **171**, **172**, and **173** were the most potent, with IC_50_ values of 49.15, 49.21, and 31.9 µM, respectively. In addition to suppressing proliferation, the compounds also triggered apoptosis, with late apoptosis reaching 28.4% for **171**. All three compounds induced caspase-3/7 activation, loss of mitochondrial membrane potential, and ROS accumulation, implicating the intrinsic apoptotic pathway. *O*-prenylation on ring B, combined with electron-withdrawing substituents (F, NO_2_, and Cl) on ring A were noted to enhance cytotoxicity, with the *ortho* (2-Cl, 2-NO_2_) or *meta* positions (3-F) being particularly important. In silico docking predicted that these chalcones interact strongly (−7 to −8.8 kcal/mol) with gastric cancer oncoproteins including MMP11, CDC6, and especially HOXA1, a transcription factor linked to poor prognosis, through H-bonding (TRP383, LYS392) and π–stacking interactions. These observations suggest HOXA1 as a plausible target of prenylated chalcones. *O*-prenylchalcones featuring electron-withdrawing groups on the B-ring may thus act as mitochondrial disruptors and apoptosis inducers in gastric cancer cells [135].


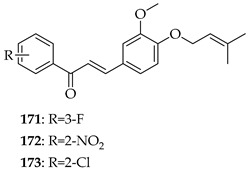


Two series (either *C*- or *O*-prenylated) trimethoxychalcones featuring Mannich base modifications were based on **3**. Screening against four cancer cell lines, Aspc-1 (pancreatic), SUN-5 (gastric), HepG2 (hepatocellular carcinoma), and HCT-116 (colon) showed that several outperformed **3**. The most active, **174** and **175**, pyrrolidinomethyl and piperidinomethyl *O*-prenylated compounds, had IC_50_ values of 2.52–5.37 μM across tested cell lines compared to **3** (IC_50_~9–16 μM). Substrate **175** in particular was broadly active (IC_50_ 2.54–10.49 μM). Introducing polar aminomethyl groups at prenylated sites thus enhanced solubility and potency, overcoming some of the bioavailability limitations of **3**. *O*-prenylated chalcones bearing cyclic amine Mannich bases were more potent than their *C*-prenylated analogues. The results confirmed the Mannich base approach as a viable strategy to optimise prenylated chalcones as anti-cancer lead compounds [136].


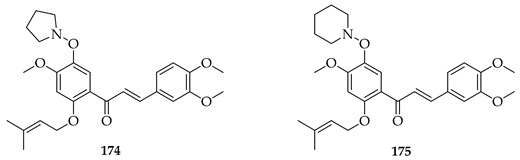


The following SAR map illustrates the structural determinants of prenylated chalcones influencing their cytotoxicity against various cancer cell lines, as described.


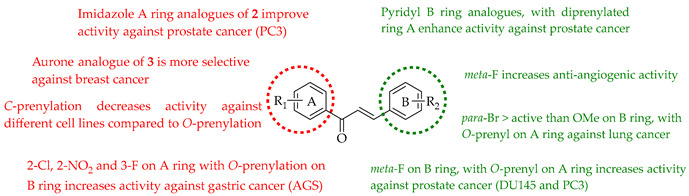


### 6.2. Anti-Inflammatory Properties of Prenylated Chalcones

Inflammation is a pathological process associated with many conditions, ranging from external infections to diseases affecting specific organs, such as the lungs (pneumonia), liver (hepatitis), and kidneys (nephritis). It reflects a complex interplay between pro-inflammatory elements and the body’s natural defence systems, which together determine how inflammation begins, develops, and eventually subsides [137]. Various groups have explored the potential of natural and synthetic prenylated chalcones as anti-inflammatory templates.

#### Anti-Inflammatory Effects of Naturally Occurring Prenylated Chalcones

Licochalcone A (**2**) can modulate anti-inflammatory processes through several means, including inhibition in lipopolysaccharide (LPS)-induced microglial cell line BV-2 phosphorylation, suggesting neuroprotective pharmacological activity [138] and also stimulation of the SIRT1/Nrf2 axis, promoting cellular protection in neuroinflammatory models such as oxygen–glucose deprivation/reperfusion (OGD/R) [139]. The ability of **2** to activate the Keap1-Nrf2 pathway has also been linked to anti-arthritic effects, suggesting a broader potential in treating oxidative stress-related inflammatory diseases, including arthritis, neurodegeneration, and autoimmune conditions [140]. Substrate **2** also inhibits the secretion of IL-1β, IL-6, and TNF-α inflammatory cytokines by downregulating TLR-4 expression and inhibiting the TLR-4/NF-κB inflammatory signalling pathway [141]. The anti-inflammatory effects of **2** were probed by targeting the Toll-like receptor 4 (TLR4) signalling pathway in an acute lung injury model induced by LPS. Substrate **2** directly interacted with the TLR4-MD2 complex, blocking LPS binding and preventing TLR4 signalling, suggesting that **2** exerts its anti-inflammatory effects through the inhibition of TLR4 signalling [142]. On ion channels in T-lymphocytes, **2** inhibited ORAI1, Kv1.3, and KCa3.1 channels in a concentration-dependent manner (IC_50_: 2.97, 0.83, and 11.21 µM, respectively), leading to reduced IL-2 secretion and T-cell proliferation. These findings suggest **2** as a potential therapeutic candidate for immune-related inflammatory diseases [143]. Substrate **2** was shown to regulate inflammation by targeting upstream proteins in LPS signalling. It directly binds to MD2, blocking LPS-induced TRIF- and MYD88-dependent TLR4 pathways, with Leu61 and Phe151 as key residues. In vivo, **2** alleviated LPS-induced acute lung injury in mice by reducing immune cell infiltration, TLR4 activation, and cytokine production [144]. Substrate **2** dose-dependently inhibited PGE_2_ release by suppressing the AA/COX pathway, reduced IL-6 and TNFα production, and blocked MAPK (p38, ERK1/2) activation. Additionally, it decreased 8-iso-PGF_2_α levels, confirming antioxidant activity [145].

Xanthohumol (**3**) was investigated against osteoarthritis by reducing IL-1β-induced inflammatory mediators and cartilage-degrading enzymes while promoting type II collagen and aggrecan. It activated Nrf2, suppressed NF-κB signalling, and delayed OA progression in vivo, suggesting **3** as a promising candidate for disease modulation [146]. In an in vitro model of mechanically stimulated cementoblasts, **3** exhibited dose-dependent effects on cell viability, with low concentrations (0.2–0.8 µM) enhancing cell viability and higher concentrations (4–8 µM) causing cytotoxicity. Also, **3** was found to significantly reduce the expression of IL-6, a key pro-inflammatory marker, in compressively stimulated cementoblasts. Furthermore, **3** re-established the phosphorylation of ERK and AKT to basal levels, which were otherwise upregulated under compressive stress. These findings were claimed to support the potential of **3** as an anti-inflammatory agent during orthodontic therapy and in treating periodontal diseases such as periodontitis [147]. Substrate **3** alleviated rheumatoid arthritis pain in a CIA mice model by reducing spinal neuroinflammation, suppressing NLRP3 inflammasome activation, and enhancing Nrf2-mediated antioxidant defences. Mechanistically, **3** bound to AMPK, promoted its phosphorylation, and mitigated mitochondrial dysfunction, thereby relieving chronic pain [148]. Substrate **3** reduced mechanical stress-induced inflammation in human periodontal ligament stem cells by lowering IL-6 expression and normalising AKT and ERK phosphorylation [149].Nine naturally occurring prenylated chalcones were synthesised and evaluated for their anti-inflammatory properties using LPS-stimulated RAW-264.7 macrophages. Among the chalcones, compounds **176** (IC_50_ = 10.41 mmol/L), **177** (IC_50_ = 9.65 mmol/L), and **122** (IC_50_ = 15.34 mmol/L) demonstrated notable activity without causing cytotoxicity. Compound **123** exhibited the strongest inhibition of NO (83.6%, IC_50_ = 4.5 mmol/L), although it had mild cytotoxicity. These findings showed that chalcones with prenyl groups at the 3- and/or 5-positions of ring A had little or no inhibitory effect, while those with prenyl substitution exclusively on ring B showed significant NO suppression without cytotoxicity [57].


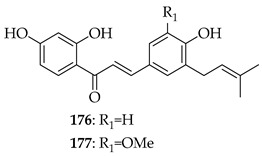


### 6.3. Anti-Microbial Properties of Prenylated Chalcones

Prenylated chalcones have gained attention for their broad-spectrum anti-microbial properties, reflecting the critical role of the prenyl moiety in enhancing lipophilicity, membrane permeability, and interactions with microbial enzymes. Unlike many conventional agents of narrow spectrum, prenylated chalcones display activity across diverse pathogens, including bacteria, fungi, and protozoan parasites, often combining direct microbicidal effects with host-protective mechanisms such as antioxidant and anti-inflammatory modulation. Studies have highlighted their ability to inhibit multidrug-resistant bacteria, potentiate frontline antibiotics such as isoniazid in tuberculosis, and suppress parasitic infections like *Leishmania infantum*, while maintaining a relatively low cytotoxicity toward mammalian cells. These findings position prenylated chalcones as versatile scaffolds for anti-microbial drug discovery, with the potential to bridge gaps in neglected infectious disease and address antibiotic resistance.

#### 6.3.1. Anti-Bacterial Properties of Prenylated Chalcones

Based on reports from the World Health Organization (WHO), bacterial resistance to commonly used antibiotics poses a significant global health challenge both now and into the future. Various strategies have been suggested to address this issue, such as targeting MDR pumps, preventing biofilm formation, and developing new antibiotics with novel mechanisms of action [150]. Chalcones have been suggested to offer the potential to serve as new therapeutic options for infectious diseases, offering a way to bypass current antibiotic resistance mechanisms [151]. Both naturally occurring and synthetic chalcones have been evaluated for anti-microbial properties.

Licochalcone A (**2**) has broad anti-microbial properties, including anti-bacterial activity against *Salmonella typhimurium,* a multidrug-resistant organism, with **2** showing minimum inhibitory concentrations (MIC) ranging from 62.5 to 1000 µg/mL. Also, **2** inhibited biofilms essential for combating persistent infections, especially with resistant strains [152]. Among various isolated prenylated phenolics from *G. inflata*, **2** had the strongest anti-bacterial activity, particularly against *Staphylococcus aureus* and *Streptococcus mutans*, with MIC values of 12.5–25 µg/mL. At concentrations corresponding to its MICs, **2** did not reduce Caco-2 cell viability, indicating relative selectivity. Emphasis was placed on the critical role of the prenyl chain in boosting anti-bacterial potency, as non-prenylated flavonoids proved much weaker (MIC > 120 µg/mL) [153]. In an investigation of the activity of **2** against the dual-species biofilms of *Listeria monocytogenes* and *S. aureus*, **2** exhibited potent inhibition, with MICs ranging from 7.5 µg/mL for both species. Also, **2** significantly reduced biofilm formation in both mono- and dual-species cultures at sub-MIC concentrations, suggesting the potential for controlling complex biofilm structures. Furthermore, **2** inhibited quorum sensing mechanisms, specifically by reducing autoinducer-2 signalling, which is critical for bacterial communication within biofilms. The study also highlighted that **2** decreased extracellular polymeric substance production, a key component of biofilm stability. The impact of **2** on biofilm formation was associated with changes in bacterial motility, hydrophobicity, and gene expression, particularly of biofilm-related genes such as *icaA* and *SarA*. When applied to beef samples contaminated with dual-species biofilms, **2** reduced viable bacterial counts by 1.1 and 1.0 log CFU/g for *S. aureus* and *L. monocytogenes*, respectively [154].

The study tested hop compounds (humulone, lupulone, and xanthohumol) against anaerobic bacteria, including *Bacteroides fragilis*, *Clostridium perfringens*, and *Clostridium difficile*. Substrate **3** showed the strongest anti-microbial effects, with MIC and minimum bactericidal concentration (MBC) values similar to conventional antibiotics. Lupulone and humulone were less effective. The results suggest xanthohumol as a potential alternative for treating infections caused by resistant *C. difficile* [155]. The anti-microbial properties of xanthohumol (**3**) and analogues were also tested against Gram-positive (*S. aureus*) and Gram-negative bacteria (*Escherichia coli*). MICs of **3** ranged from 31.25 to 500 µg/mL, depending on the bacterial strain, but were particularly active against Gram-positive species. The study also compared **3** with several analogues, such as xanthohumol D (**147**), and found that **3** was more potent. Moreover, **3** demonstrated biofilm inhibition, reducing biofilm formation in *S. aureus* and *E. coli* [156].

Isobavachalcone (**80**) was active against MSSA and MRSA, with MICs of 1.56 µg/mL for MSSA and 3.12 µg/mL for MRSA, comparable to tetracycline. It also inhibited *Streptococcus sanguinis* and *S. mutans* at 3–6 µg/mL while being inactive against *Pseudomonas aeruginosa* and *Klebsiella pneumoniae* (MIC > 400 µg/mL), ascribed to the permeability barrier of Gram-negative organisms. Substrate **80** suppressed MSSA and MRSA biofilm formation by >50% at 0.78 µg/mL, with an efficacy comparable with vancomycin (0.74 µg/mL). It also had moderate antimycobacterial activity (MIC = 62.5 µg/mL) against *Mycobacterium tuberculosis*, *M. avium*, and *M. kansasii*. Mechanistic assays revealed that **80** disrupted bacterial membranes in *Bacillus subtilis*, causing a loss of integrity in ~75% of cells. This was deemed consistent with the prenyl-driven insertion into lipid bilayers. Importantly, **80** showed no cytotoxicity to human keratinocytes (HaCaT) up to 25 µg/mL, a concentration ~20-fold higher than its anti-MRSA MIC. The prenyl substituent was considered to enhance membrane affinity and selectivity toward Gram-positive organisms, thus explaining the strong biofilm inhibition and low mammalian toxicity, while also accounting for relative inactivity against Gram-negative species [157]. In a more recent study utilising *S. aureus*, MRSA, *E. coli*, and *P. aeruginosa*, **80** showed consistently strong Gram-positive activity (MIC = 5 μM against *S. aureus*), corroborating the earlier results, but was inactive against MRSA and Gram-negatives. The prenyl chain on the A-ring and free hydroxyl groups were deemed essential for activity [158].

Broadening the spectrum of activity of **80** was the aim of a hybrid pharmacophore approach, which conjugated **80** with 3-hydroxy-pyridin-4(1*H*)-ones, iron-chelating moieties recognised by bacterial iron uptake systems. Eight conjugates were synthesised, and under iron-limited conditions, they showed 8–32-fold lower MICs and up to 177-fold lower IC_50_s against *P. aeruginosa* PAO1 and MDR strains than parent **80**. The lead compound, **178**, displayed MIC = 16 µg/mL and IC_50_ = 1.45 µg/mL versus PAO1, while also protecting *C. elegans* from lethal PAO1 infection (55% survival at 60 h with twice the MIC dose). SAR analysis indicated that attachment at C-6 of the hydroxypyridinone moiety improved potency over C-2 substitution, that short alkyl linkers outperformed longer chains, and that amide linkers enhanced activity against Gram-negative organisms compared to triazoles. Conjugate **178** damaged PAO1 membranes and reduced metabolic activity, similar to the mode of action of **80** against Gram-positive organisms. Conjugate **178** was also less cytotoxic to mammalian Vero cells than **80**, possibly because siderophore conjugation reduced host uptake. The designed conjugate was thus deemed to have successfully incorporated the anti-bacterial pharmacophore alongside a siderophore tag, enabling Gram-negative entry without the loss of activity [159].


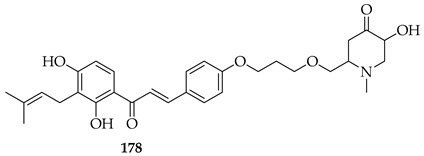


Paratocarpin E (**107**), another natural chalcone, was found to inhibit the growth of two different Gram-positive bacteria, *B. subtilis* and *S. aureus*, with MIC values of 6.25 and 25 μg/mL, respectively [74].

The di-*O*-prenylated chalcone **179**, bearing two arginine residues, showed potent activity against Gram-positive bacteria, including MRSA, comparable to vancomycin. It exhibited low haemolytic and cytotoxic effects, high membrane selectivity, and rapid bactericidal action. It disrupted bacterial membranes, causing cell death, and demonstrated in vivo efficacy against *S. aureus* in a murine keratitis model [160].


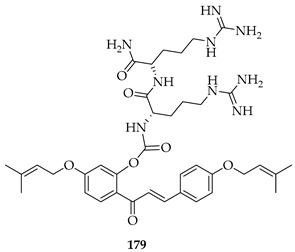


#### 6.3.2. Anti-Fungal Properties of Prenylated Chalcones

The increasing prevalence of fungal infections, especially those caused by resistant strains, poses a significant global health challenge. Resistance hampers clinical management, particularly in immunocompromised patients [161]. As a result, there is a growing need for new therapeutic approaches, including the development of anti-fungal agents with novel mechanisms of action. Chalcones, both naturally occurring and synthetic, have demonstrated promising anti-fungal properties. Their ability to disrupt fungal cell membranes and interfere with key biological processes makes them potential candidates for combating resistant fungal infections. The anti-fungal activity of prenylated chalcones is attributed to their ability to disrupt the fungal cell membrane, inhibit enzyme activity, and interfere with various cellular processes essential for fungal growth. Prenylated chalcones have shown activity against a range of pathogenic fungi, including *Candida* species and dermatophytes, making them promising broad-spectrum candidates. Their structure allows for the facile modulation of activity, potentially overcoming resistance mechanisms.

The anti-fungal effects of Licochalcone A (**2**) were evaluated against *C. albicans* biofilms in vitro and in vivo. Substrate **2** significantly inhibited biofilm growth at 625 μM (10 x MIC) by reducing colony formation and biofilm dry weight while decreasing proteolytic enzyme activity, a key virulence factor. Topical treatment in a mouse model resulted in a substantial reduction in fungal load, confirmed by bioluminescence imaging and the microbiological analysis of infected tissue, with less fungal invasion observed in histopathological samples. Additionally, **2** had minimal toxicity to oral fibroblasts in a co-culture model and modulated the immune response by reducing pro-inflammatory cytokines IL-1α and IL-1β and increasing IL-10. Overall, **2** was held to offer an effective and low-toxicity treatment for oral candidiasis [162].

The potential of **2** in treating *Aspergillus fumigatus*-induced fungal keratitis, a severe eye infection that can lead to blindness, was evaluated. Substrate **2** was shown to disrupt fungal biofilms, to inhibit fungal growth and adhesion, and to induce morphological changes in the hyphal structure of *A. fumigatus*. Additionally, **2** compromised the integrity of the fungal cell membrane, cell wall, and mitochondrial structure. In a mouse model, treatment with **2** alleviated the severity of keratitis by reducing neutrophil infiltration, decreasing fungal load, and lowering pro-inflammatory corneal cytokine levels. At the cellular level, **2** activated the Nrf2/HO-1 signalling pathway in human corneal epithelial cells, demonstrating anti-inflammatory properties [163].

Apart from efficacy against human pathogens, the anti-fungal activity of both hop leaf extracts and isolated **3** were evaluated against plant pathogens. Both extracts and **3** caused the dose-dependent inhibition of triazole-sensitive and -resistant strains of *Venturia inaequalis*, with **3** showing stronger activity. The MIC of **3** was 12.5 µg/mL for the triazole-sensitive strain and 25 µg/mL for the triazole-resistant strain, while crude hop extracts had MICs of 50 µg/mL and 100 µg/mL, respectively. Also, **3** significantly reduced conidial germination and mycelial biomass, achieving a 70–85% inhibition at 50 µg/mL after 72 h. The results supported the application of **3** in the management of apple scab. Among 16 novel prenylated chalcones evaluated for their anti-fungal properties against *Sclerotium rolfsii* and *Fusarium oxysporum*, **180** exhibited the strongest anti-fungal activity, with ED_50_ values of 25.02 mg/L against *S. rolfsii* and 31.87 mg/L against *F. oxysporum*. Molecular docking studies targeting *EF1α* and *RPB2* gene products in *S. rolfsii* and the FoCut5a protein in *F. oxysporum* supported the interactions of the chalcones with these targets, reflected in the binding energies ranging from −38.35 to −26.68 kcal/mol for *S. rolfsii* and −43.40 to −23.84 kcal/mol for *F. oxysporum*. Further docking studies confirmed that the chalcones formed stable complexes with the respective proteins. The same group reported on a further series of prenylated chalcones against the same fungal pathogens, with additional SAR analysis and docking studies. Among these compounds, **181** showed the highest activity against *S. rolfsii* (ED_50_ = 23.28 mg/L), while **182** was the most effective against *F. oxysporum* (ED_50_ = 25.70 mg/L). Docking studies targeted DNA-directed RNA polymerase (*RPB2* gene) in *S. rolfsii* and cutinase (*FoCut5a* gene) in *F. oxysporum*. The results revealed favourable binding energies, ranging from −31.05 to −19.36 kcal/mol for *S. rolfsii* and from −38.95 to −26.50 kcal/mol for *F. oxysporum*, suggesting strong inhibitory potential [164].


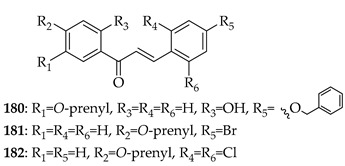


The following map represents the SAR of prenylated chalcones against bacterial and fungal pathogens, highlighting key prenylation sites associated with improved anti-microbial potency.


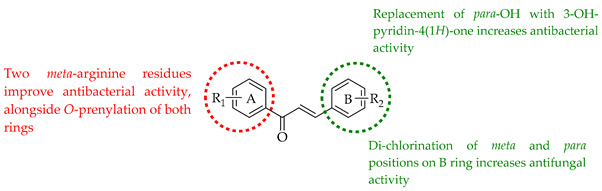


### 6.4. Anti-Leishmanial Properties of Prenylated Chalcones

Millions of people worldwide, mostly in tropical and subtropical areas, suffer from leishmaniases, a group of diseases caused by protozoan parasites in the genus *Leishmania*. Humans contract the parasite through the bite of infected female phlebotomine sandflies [165]. Skin, mucocutaneous, and visceral (kala-azar) leishmaniases are the three primary types; visceral leishmaniasis is the most serious and can be fatal [166]. Currently available anti-leishmanials include pentavalent antimonials, amphotericin B, miltefosine, and paromomycin, which possess significant toxicities, require protracted treatment periods, and are subject to increasing resistance [167].

Licochalcone A (**2**) has long-established anti-leishmanial activity [168]. When tested against *L. amazonensis* and *L. infantum*, **2** showed high in vitro activity, with IC_50_ values as low as 3.88 µM for promastigotes and 29.58–36.84 µM for amastigotes. In infected hamsters, **2** (at 50 mg/kg for 8 days) reduced parasite loads by 43.67% in the liver and 39.81% in the spleen. These findings support the potential of licochalcone-type constructs as candidates for treating visceral leishmaniasis [169].

Xanthohumol (**3**) was evaluated in *L. amazonensis* promastigotes and amastigotes (LaP/LaA) and compared to its effects on mouse peritoneal macrophages and the J774A.1 macrophage cell line (J774). Additional mechanistic analyses were performed using *L. tarentolae* promastigotes (LtP) and isolated mitochondrial fractions. Substrate **3** showed inhibitory activity against LaA with an IC_50_ of 7 µM. In contrast, **3** had an IC_50_ of 70 µM in macrophages. Notably, **3** suppressed oxygen consumption in LtP, with inhibition linked to **3** targeting the mitochondrial electron transport chain at complex II/III [170].

Further naturally occurring chalcones were tested for their ability to combat intracellular amastigotes of *L. amazonensis* and to target *Trypanosoma cruzi*, the causative agent of Chagas disease. The prenylated chalcones isobavachalcone (**80**), 4-hydroxylonchocarpine (**183**) and lonchocarpine (**184**), were inactive alone against *L. amazonensis*, although **184** was effective against *T. cruzi*. The anti-parasitic activity of various mixtures of these compounds in different ratios (3:1, 1:1, and 1:3) was also assessed. Compounds **80** and **183**, although individually inactive against *T. cruzi*, showed trypanocidal effects when combined. The activity of compound **183** (IC_50_ = 13.63 µg/mL) improved when mixed with compound **80** in 1:1 (IC_50_ = 10.01 µg/mL) and 3:1 (IC_50_ = 7.78 µg/mL) ratios [171].


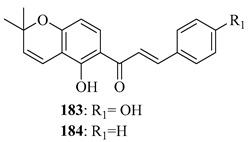


A series of prenylated chalcones featuring electron-attracting or electron-donating substituents on rings A and B was synthesised. Out of 25 compounds tested against *L. mexicana* promastigotes, eleven exhibited selectivity with minimal toxicity to mammalian macrophages. Among them, compounds **185**, **186**, and **187** demonstrated the strongest anti-leishmanial activity. Interestingly, **185** and **186**, possessing a *meta* substituent on ring B, suggest that substitution pattern influences activity. Substrate **186** showed the highest potency with an IC_50_ of 4.57 µM and a SI exceeding 297.62. SAR analysis indicated that anti-leishmanial effectiveness improves with an *O*-prenyl group on ring A and a *meta*-nitro group on ring B. Additionally, this study presented the first homology model of *L. mexicana* fumarate reductase combined with a blind docking strategy. Docking results suggested that chalcones interact with fumarate reductase at multiple binding sites. Compound **185** bound effectively at the fumarate site, potentially creating steric hindrance to disrupt normal substrate interaction. These findings highlighted fumarate reductase as a validated target in the development of novel anti-leishmanials [172].


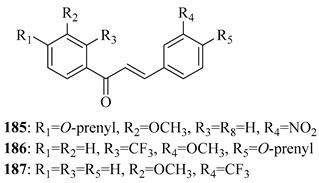


Another series of chalcones bearing prenyloxy or geranyloxy substituents was synthesised to probe how substitution patterns affect leishmanicidal and trypanocidal activity. Substituents were varied on either A or B rings of the chalcone scaffold. Compounds **188** and **189** exhibited the strongest parasiticidal effects against both *L. mexicana* and *T. cruzi*, while **190** and **191** showed species-specific activity: **190** against *L. mexicana* and **191** against *T. cruzi*. Compound **188** had the highest SI for both parasites (80.9 for *L. mexicana* and 75.1 for *T. cruzi*), attributed to lower cytotoxicity compared to its structural isomer, **189**. Also, **191** had a higher SI value for *T. cruzi* than the standards nifurtimox and benznidazole. Morphological studies showed that exposure to **188** reduced promastigote cell density in both parasite species. SAR analysis confirmed that both the nature and position of substituents were determinants of biological activity and selectivity. Although a precise mechanism of action was unclear, the findings supported further investigation, particularly of compound **188** [173].


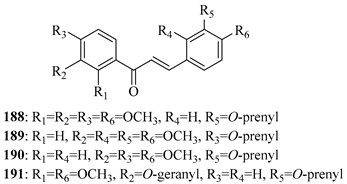


A series of thirty-one novel chalcone derivatives was tested for activity against *L. donovani* promastigotes. Sixteen showed anti-leishmanial effects with IC_50_ values ranging from 3.0 to 21.5 µM, with low toxicity toward mammalian cells. Of these, **200** demonstrated strong inhibitory effects on both promastigote and intracellular amastigote forms, along with high SIs. It also inhibited the growth of other *Leishmania* species, including *L. tropica*, *L. major*, and *L. infantum*. Additionally, **200** showed a strong binding affinity to trypanothione reductase, a key parasite enzyme, with sub-micromolar inhibitory potency. These combined features were considered to position compound **200** as a promising antileishmanial lead [174].


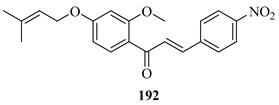


The following SAR map depicts the relationship between the prenylation pattern of chalcones and their activity against *Leishmania* parasites, emphasising key structural features linked to enhanced leishmanicidal effects.


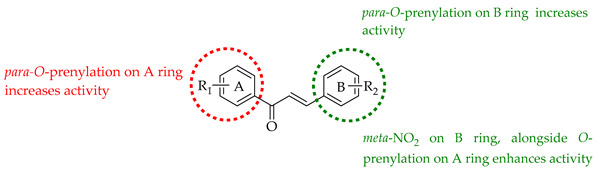


### 6.5. Anti-Malarial Properties of Prenylated Chalcones

Malaria, caused by infection with *Plasmodium* species, notably *P. falciparum*, is one of the deadliest infections, with over half a million deaths and >260 million cases annually across the globe [175]. Several common anti-malarial drugs such as chloroquine have limited effectiveness due to the development of drug-resistant strains, and the discovery of new potent and affordable treatments is needed [176]. Chalcones have demonstrated potential as a structural class imbued with anti-malarial activity, particularly through inhibition of malarial cysteine proteases [177]

Based on the literature data for anti-malarial activity of prenylated chalcones, seven synthesised chalcones, including prenylated and allylated derivatives, were assessed against the chloroquine-sensitive *P. falciparum* 3D7 strain. Prenylated chalcone **193** showed the best IC_50_ value of 1.08 μg/mL against this strain (1.37 μg/mL based on QSAR analysis). Docking simulations revealed that **193** was well-positioned in the active site of *P. falciparum* dihydrofolate reductase-thymidylate synthase, enabling H-bonding interactions with Ala16, Ser108, and Ile164 and π-bond interactions with Trp48 and Phe58. Phe58 interaction could interrupt catalysis reactions in the thymidylate cycle, thus preventing deoxythymidine monophosphate production and DNA synthesis. SAR analysis emphasised that prenyl substitution on the A-ring-enhanced binding affinity and anti-malarial potency [178].


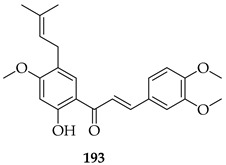


### 6.6. Anti-Diabetic Properties of Prenylated Chalcones

Diabetes, characterised by high blood glucose levels, is a growing global health issue. While existing medications help manage the condition, they often have limitations. Prenylated chalcones, a class of natural compounds, show promise as anti-diabetic agents. They can regulate blood sugar, improve insulin sensitivity, and inhibit enzymes involved in glucose metabolism, offering a potential natural alternative for diabetes management [179].

The effects of Licochalcone A (**2**) on Type 2 diabetes mellitus (T2DM) in mice, induced by a high-fat diet and streptozocin, was investigated. Substrate **2** significantly reduced insulin resistance, blood glucose, and serum lipid levels. It also alleviated T2DM symptoms, such as excessive drinking, eating, urination, and weight loss. Substrate **2** improved oral glucose tolerance, pancreatic damage, and liver enlargement. Network pharmacology revealed that its effects were mediated by regulating the insulin signalling pathway, particularly through the PI3K/Akt pathway, which helped improve insulin sensitivity and protect islet cells. The high-dose group of **2** showed the best results, suggesting its potential as a nutritional agent for T2DM treatment [180].

The effects of xanthohumol (**3**) on α-amylase and α-glucosidase, key enzymes in carbohydrate metabolism and glucose absorption were explored. Using kinetics, multi-spectral, and molecular docking methods, the results showed that **3** inhibited both enzymes in a reversible mixed-type manner, with IC_50_ values of 71.07 ± 5.82 μM for α-amylase and 32.58 ± 3.11 μM for α-glucosidase. Multi-spectral analyses showed that the binding of **3** caused conformational changes and microenvironment alterations in the enzymes, inhibiting their activities [181].

The prenylated chalcone 4-hydroxyderricin (**88**) was demonstrated to prevent postprandial hyperglycaemia by promoting GLUT4 translocation in skeletal muscle. In L6 myotubes, **88** increased glucose uptake in a dose-dependent manner, with **88** active at 0.1 μM. Mechanistic studies showed that **88** activated the LKB1/AMPK signalling cascade, as siRNA knockdown or treatment with an AMPK inhibitor abolished their glucose-lowering effects. Substrate **88** acted independently of insulin and JAK/STAT signalling, distinguishing it from insulin mimetics. In vivo, the oral administration of **88** (10 and 50 mg/kg) significantly suppressed glucose spikes in ICR mice after an oral glucose tolerance test. SAR interpretation within the study emphasised the importance of the prenyl group and hydrophobic substitution patterns, with **88** bearing a methoxy group that improved absorption and exhibited greater potency at lower doses. This informed how prenylation combined with optimal hydrophilic balance enhanced bioavailability and AMPK activation, and the compound was considered a promising dietary agent for metabolic syndrome and prevention of type 2 diabetes [182].

### 6.7. Neuroregenerative Activity

The prenylated chalcones morachalcone D (**194**) and morachalcone E (**195**) were evaluated for their neuroprotective effects in HT22 hippocampal neurons. Both compounds were non-toxic up to 50 μM. Substrate **194** potently prevented glutamate-induced oxytosis and erastin-induced ferroptosis, with EC_50_ values of 30.56 μM and 33.73 μM, respectively, while **195** showed only weak protection (<25% viability rescue). Substrate **194** was demonstrated to reduce ROS generation, preserve intracellular glutathione, inhibit Fe^2+^ accumulation, and upregulate antioxidant defence genes (*GPx4*, *CAT*, *SOD2*, *Nrf2*, *HMOX1*, and *SLC7A11*). SAR analysis noted the effect of the prenylation pattern on activity; while **194** has a 2,3-dihydroxy-3-methylbutyl side chain at C-3′, **195** has a furan ring and, consequently, one less hydroxyl, reducing antioxidant capacity. Hydroxylated prenyl chains were considered advantageous for antioxidant and ferroptosis-inhibitory activity. Overall, **194** was held as a promising lead for neuroprotection against oxidative stress and ferroptosis-related neurodegeneration [183].


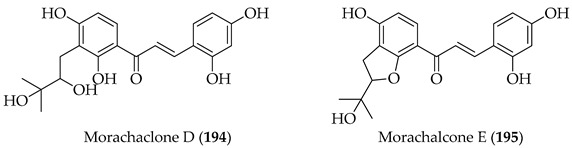


Prenyl- and pyranochalcones were compared for their ability to induce neuronal differentiation of adult neural stem cells using a dual luciferase reporter assay under the human doublecortin X (DCX) promoter. The pyranochalcone xanthohumol C (**131**) was the most active, producing a differentiation-inducing activity (DIA) of 5.55 ± 1.09, surpassing both retinoic acid/valproic acid (3.22 ± 1.02) and the parent **3** (DIA 1.56 ± 0.49). Other pyranoflavonoids consistently outperformed their prenyl analogues, establishing the pyrano ring as a critical structural motif for the induction of neurogenesis. SAR interpretation emphasised how pyranisation of the prenyl group enhanced neuronal differentiation activity, while B-ring hydroxylation favoured activity over methoxy substitution. Overall, these pyranochalcones were considered promising neuroregenerative leads capable of overriding CNS growth inhibitors and stimulating neurogenesis [184].

## 7. Pharmacokinetic and Druggability Concerns

Despite an abundance of the literature data cited here and elsewhere, with notable exceptions already discussed (Section 1), few prenylated chalcones have progressed to clinical stages of development. The reasons for this are multi-factorial. The innate reactivity of the α-β-unsaturated system and its identity as a Michael acceptor renders specificity a concern, with the enone system considered by some to represent a PAINS scaffold [185]. However, despite being accepted as a key pharmacophoric element among chalcones in general, there may not be an absolute requirement for this potentially non-specific functionality. This is supported by several papers, whereby activity was retained in dihydrochalcone analogues [186]. Concerns regarding toxicity, also linked to non-specificity, have not been borne out in most studies reported in this review, with selectivity indices broadly favourable. In addition, as discussed in a recent review [187] on the druggability of chalcones in general, pharmacokinetic and pharmacodynamic properties of chalcones may be sub-optimal, attributed to poor water solubility and potential for drug interactions through the inhibition of efflux pumps including P-glycoprotein. Data on the bioavailability of prenylated chalcones in human subjects remains scarce, however, yet available information supports challenges in the druggability of these compounds. In a study of five healthy volunteers who ingested 43 mg of either native or micellar xanthohumol (**3**), conjugated metabolites were determined using LC–MS/MS without enzymatic deconjugation. Free **3** represented <1% of plasma levels, confirming rapid and near-complete phase II metabolism. The dominant metabolites were **3**-7-*O*-glucuronide and **3**-4′-*O*-sulfate, with additional glucuronides and mixed sulfate-glucuronides of isoxanthohumol and 6- and 8-prenylnaringenins. Compared to native **3**, the micellar formulation achieved 5-fold higher AUC and >20-fold higher Cmax for the **3**-7-*O*-glucuronide, with Tmax ~0.8 h (vs. 2.1 h for 3). Pharmacokinetic curves revealed double maxima for conjugates, suggesting enterohepatic recirculation. After 7 days of repeated dosing (3 × 43 mg/day), metabolite accumulation was minimal, but fasting plasma concentrations trended higher with micellar **3**. These results suggested that the prenyl group makes **3** highly lipophilic, limiting free absorption, and that conjugation at hydroxyl groups (mainly glucuronidation at 7-OH) produces stable, circulating metabolites. Micellization improved solubility and protected against gastric cyclization to isoxanthohumol, explaining the superior systemic exposure and highlighting how formulation considerations are important in the advancement of these compounds [188]. However, despite these concerns, the variety of synthetic methodologies discussed in Section 5 and the ease of diversification of the chalcone scaffold are advantageous in the rapid screening of broad panels of chalcone derivatives. We have noted throughout the paper how selective design strategies have been utilised to optimise the modulation of the hydrophilic–lipophilic balance through optimal hydroxylation, halogen substitution, and how the nature and extent of prenylation is critical to the activity of these intriguing compounds. Such structural optimisation, coupled with formulation strategies such as the use of appropriate excipient profiles and nanotechnological drug delivery systems, offer significant potential in successful translational applications.

## 8. Conclusions

This review highlights the growing significance of prenylated chalcones as a unique and versatile class of bioactive compounds. We highlighted the most recently isolated prenylated chalcone natural products and discussed in depth the synthetic approaches for prenyl insertion, from simpler approaches including direct *C*- and *O*-prenylations to the more complex, such as prenyl rearrangements and cyclisations. From a synthetic perspective, the increasing availability of diverse methodologies provides chemists with powerful tools to generate analogues and probe structure–activity relationships. While these approaches enable access to diverse scaffolds, challenges remain, including regioselectivity, stereocontrol, and scalability, which may hinder broader exploration, particularly of complex, multi-prenylated frameworks. In particular, rearrangement-based routes, such as those employed in the synthesis of Licochalcone A, often suffer from low yields, highlighting significant limitations when compared to the efficiency required for industrial-scale production. Therefore, when compared to their non-prenylated counterparts, while the structural complexity introduced by prenylation can enhance biological activity, it can also complicate synthesis and restrict large-scale accessibility for translational study. Another important consideration is sustainability: isolation from natural sources is inefficient and laborious, and several synthetic methods rely on hazardous reagents or energy-intensive conditions, including thermal reactions in restricted solvents and the use of specialised reactors, which run counter to the principles of green chemistry. While microwave syntheses are promising, they may not be readily amenable to scale-up. Developing more environmentally benign, atom-economical, and resource-efficient strategies will be essential to make prenylated chalcone syntheses both practical and sustainable.

Wide-ranging pharmacological effects of these interesting and diverse structures were also explored, highlighting strong anti-cancer, anti-inflammatory, anti-microbial, anti-leishmanial, and anti-malarial properties observed across numerous studies, which reflect the broad therapeutic potential of this compound class. Demonstration of potential synergistic actions, notably against anti-cancer targets, could potentially enhance therapeutic activity while reducing toxicity through dose reduction. The incorporation of prenyl groups consistently enhances pharmacological profiles by improving lipophilicity, membrane permeability, and target affinity, while activities are also influenced by hydroxylation, incorporation of heterocyclic rings, and halogenation patterns. Insights from SAR analyses underscore how specific modifications can profoundly influence biological outcomes. Despite these interesting observations, important drawbacks remain: many published properties are to date only supported by in vitro data, while systematic evaluations of pharmacokinetics, toxicity, and in vivo efficacy remain scarce. However, notable examples of translational success are exemplified by those few naturally occurring prenylated chalcones that have been assessed in clinical trials.

To successfully progress prenylated chalcones as viable lead compounds, an integrated strategy is critical. On the synthetic side, innovation should focus not only on efficiency and selectivity but also on the deliberate development of sustainable, green chemistry-driven approaches, including biocatalytic and chemoenzymatic strategies, to reliably generate analogues for testing. From a biological perspective, future work must expand beyond preliminary activity screens to include mechanistic investigations, comprehensive pharmacokinetic and toxicology studies, and robust in vivo models, as all of these aspects are under-explored. Without such data, the therapeutic relevance of prenylated chalcones cannot be fully assessed. Ultimately, the synergy between environmentally conscious synthetic innovation and systematic biological evaluation will determine whether prenylated chalcones progress from hypothetically interesting molecular scaffolds to viable therapeutic agents.

## Figures and Tables

**Table 1:** Naturally occurring prenylated (& geranylated) chalcones, isolated from July 2020 until now.

Chemical Structure	Name	N°	Origin	Ref
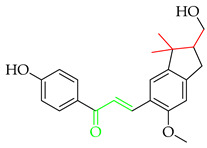	Licochalcone L	**6**	*Glycyrrhiza inflata*	[32]
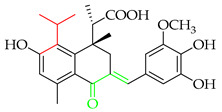	Gagone 1	**7**	*Mansonia gagei*	[33]
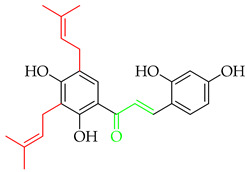	Sophotonin I	**8**	*Sophora tonkinensis*	[34]
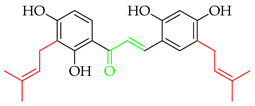	Sophotonin J	**9**	*Sophora tonkinensis*	[34]
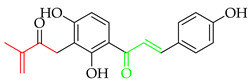	Erosusone	**10**	*Pachyrhizus erosus*	[35]
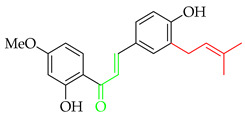	Licoagrochalcone A 4′-methylether	**11**	*Anthyllis vulneraria*	[36]
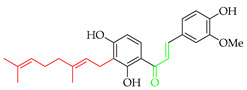	Cajavolubone A	**12**	*Cajanus volubilis*	[37]
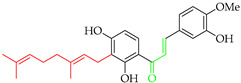	Cajavolubone B	**13**	*Cajanus volubilis*	[37]

**Table 2:** Enzymatic prenylation strategies to elaborate the chalcone core of **14**–**23**.

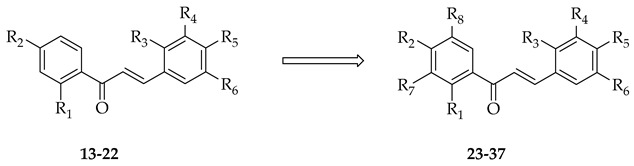			
Entry	Substituents (R_1_–R_8_ = H Unless Otherwise Stated)	Prenyltransferase	Product (R_1_–R_8_ = H Unless Otherwise Stated)
1	**14**: R_3_ = OH	AnaPT	**24**: R_3_ = OH, R_6_ = prenyl
2	**15**: R_3_ = OH, R_4_ = OCH_3_		**25**: R_3_ = OH, R_4_ = OCH_3,_ R_6_ = prenyl
3	**16**: R_5_ = OH	AtaPT	**26**: R_5_ = OH, R_6_ = prenyl
4	**17**: R_4_ = OH		**27**: R_5_ = prenyl, R_6_ = OH
			**28**: R_3_ = prenyl, R_5_ = prenyl, R_6_ = OH
5	**18**: R_2_ = OH, R_3_ = Cl		**29**: R_2_ = OH, R_3_ = Cl, R_8_ = prenyl
6	**19**: R_2_ = OH, R_5_ = F		**30**: R_2_ = OH, R_5_ = F, R_8_ = prenyl
7	**20**: R_2_ = OH		**31**: R_2_ = OH, R_8_ = prenyl
8 9	**21:** R_1_ = OH, R_5_ = OCH_3_ **22**: R_1_,R_2_,R_5_,R_6_ = OH	AnaPT	**32**: R_1_ = OH, R_5_ = OCH_3,_ R_8_ = prenyl
			**33**: R_1_,R_2_,R_5_,R_6_ = OH, R_4_ = prenyl
			**34**: R_1_,R_2_,R_6_ = OH, R_5_ = *O*-prenyl
			**35**: R_1_,R_2_,R_6_ = OH, R_4_ = prenyl, R_5_ = *O*-prenyl
10	**23**: R_1_,R_2_,R_5_ = OH		**36**: R_1_,R_2_,R_5_ = OH, R_3_ = prenyl
			**37**: R_1_,R_2_,R_5_ = OH, R_3_ = prenyl, R_7_ = prenyl
			**38**: R_1_,R_2_ = OH, R_3_ = prenyl, R_5_= *O*-prenyl

**Table 3:** Enzymatic geranylation of chalcones **14**–**23** with various prenyltransferases.

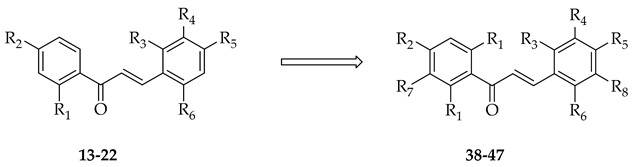		
Entry	Substrate (R_1_–R_6_ = H Unless Otherwise Stated)	Product (R_1_–R_6_ = H Unless Otherwise Stated)
1	**14**: R_3_ = OH	**39**: R_3_ = OH, R_8_ = Geranyl
2	**15**: R_3_ = OH, R_4_ = OCH_3_	**40**: R_3_ = OH, R_4_ = OCH_3_, R_8_ = Geranyl
		**41**: R_3_ = OH, R_4_ = OCH_3_, R_8_ = (reverse) Geranyl
3	**16**: R_5_ = OH	**42**: R_4_ = Geranyl, R_5_ = OH
4	**18**: R_2_ = OH, R_3_ = Cl	**43**: R_2_ = OH, R_3_ = Cl, R_7_ = Geranyl
5	**19**: R_2_ = OH, R_5_ = F	**44**: R_2_ = OH, R_5_ = F, R_7_ = Geranyl
		**45**: R_2_ = *O*-Geranyl, R_5_ = F
6	**20**: R_2_ = OH	**46**: R_2_ = OH, R_7_ = Geranyl
7	**22**: R_1_,R_2_,R_4_,R_5_ = OH	**47**: R_1_,R_2_,R_4_,R_5_ = OH, R_8_ = Geranyl
8	**23**: R_1_,R_2_,R_5_ = OH	**48**: R_1_,R_2_,R_5_ = OH, R_4_ = Geranyl

**Table 4:** Impact of base selection on *O*-prenylation of **49** and **52** in alkaline media.

Substrate	Entry	R	Reagents and Conditions	Product	R	Yield	Ref
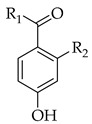	1	**49**: R_1_ = CH_3,_ R_2_ = OH	3,3-dimethylallyl bromide, DBU, acetone, RT, and 24 h.	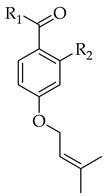	**50**: R_1_ = CH_3_ R_2_ = OH	76%	[47]
	2	**52**: R_1_ = H, R_2_ = H	3,3-dimethylallyl bromide, NaH, DMF, RT, and 1 h.		**53**: R_1_ = H R_2_ = H	84%	[48]
	3	**49**: R_1_ = CH_3,_ R_2_ = OH	3,3-dimethylallyl bromide, KOH, water, RT, and 12 h.		**50**: R_1_ = CH_3_ R_2_ = OH	2%	[49]

DMF = *N*,*N*-Dimethylformamide.

**Table 5:** Catalytic *O*-prenylation in alkaline media.

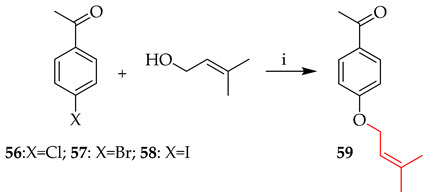				
Entry	X	Reagents and Conditions (i)	Yield	Ref
1	**56**: X = Cl	NiBr_2_·3H_2_O, 4,4′-di-*tert*-butyl-2,2′-bipyridyl, DBU, PhSiH_3_, toluene, 120 °C, and 24 h.	89%	[52]
2	**57**: X = Br		85%	
3	**57**: X = Br	NaI, CuI, Cs_2_CO_3_, DMEDA, 1,10-phenanthroline, toluene, 110 °C, and 24–36 h.	77%	[53]
4	**58**: X = I	CuI, Cs_2_CO_3_, 1,10-phenanthroline, toluene, 110 °C, and 24–36 h.	89%	

DMEDA= *N*,*N*′-Dimethylethylenediamine.

**Table 6:** *C*-prenylation of substituted benzaldehydes and acetophenones.

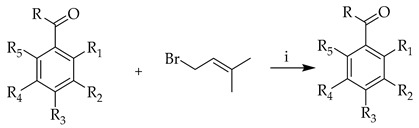					
Entry	Substrate (R–R_4_)	Reagents and Conditions (i)	Product (R–R_4_)	Yield	Ref
1	**68**: R = R_2_ = R_4_ = R_5_ = H, R_1_ = R_3_ = OH	Al_2_O_3_, THF, RT, and 72 h.	**71**: R = R_4_ = R_5_ = H, R_1_ = R_3_ = OH R_2_ = prenyl	21%	[56]
2	**68**	BaO-Al_2_O_3_ (1:1), Et_2_O, RT, and 72 h.	**71**	20%	
3	**68**	ZnO-Al_2_O_3_ (1:1), Et_2_O-hexane (1:3), RT, and 72 h.	**71**	18%	
4	**68**	KOH, H_2_O, RT, and 4 h.	**72**: R = R_2_ = R_5_ = H, R_1_ = R_3_ = OH, R_4_ = prenyl	30%	
5	**52**	1 mol/L NaOH, 0 °C-RT, and 2 h.	**63**: R = R_1_ = R_2_ = H, R_3_ = OH, R_4_ = prenyl	24%	[57]
6	**69**: R = R_1_ = R_4_ = R_5_ = H, R_2_ = OMe, R_3_ = OH	1 mol/L NaOH, 0 °C-RT, and 2 h.	**73**: R = R_1_ = R_4_ = H, R_2_ = OMe, R_4_ = prenyl	18%	
7	**70**: R = CH_3_, R_1_ = R_3_ = R_5_ = OH, R_2_ = R_4_ = H	DBU, THF, RT, and 12 h.	**74**: R = CH_3_, R_1_ = R_3_ = R_5_ = OH, R_2_ = prenyl	38%	[58]

**Table 7:** Influence of reaction conditions on Suzuki coupling of **81**.

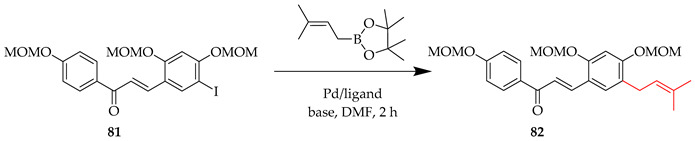				
Substrate	Temperature °C	Pd/Ligand	Base	Yield
**81**	70 °C	Pd(PPh_3_)_4_	CsF	35%
			KOAc	30%
			Cs_2_CO_3_	30%
			K_3_PO_4_	25%
		Pd(OAc)_2_	Cs_2_CO_3_	58%
		PdCl_2_(dppf)		84%
		PdCl_2_(PPh_3_)_4_		53%
	80 °C	PdCl_2_(dppf)		55%

**Table 8:** Influence of reaction conditions on the *O*- to *C*-prenyl rearrangement of **90**.

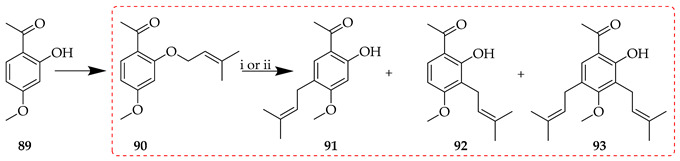				
Entry	Conditions	Yield (%)		
		91	92	93
1	(i) Montmorillonite K10, DCM, 0 °C, and 0.5 h	53	25	7
2	(ii) Florisil, toluene, reflux, and 2 h	27	32	0

**Table 9:** Influence of reaction conditions on the [3,3]-sigmatropic rearrangement of **98**.

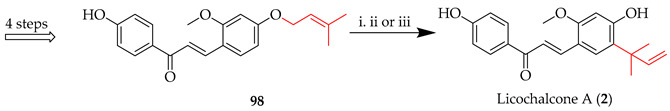		
Entry	Conditions	Yield (%)
1	(i) EtOH-H_2_O (4:1, *v*/*v*), MW (300W), 210 °C, and 1.5 h	86
2	(ii) EtOH-H_2_O (4:1, *v*/*v*), Ace pressure tube, 130 °C, and 24 h	82
3	(iii) TMSN = C(Me)OTMS, PhNEt_2_, stainless-steel bomb, 185 °C, and 5 h	56

**Table 10:** Influence of reaction conditions on the *O*-prenyl cyclisation of **90**.

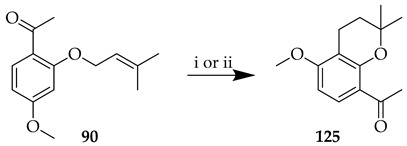		
Reaction	Conditions	Yield (%)
i	Montmorillonite K10 (1 wt equiv), DCM, 0 °C, and 0.5 h	0
ii	Florisil (10 wt equiv), toluene, reflux, and 2 h	8

## Data Availability

Data sharing is not applicable.

## Abbreviations

The following abbreviations are used in this manuscript.

AnaPT	*Aspergillus niger* aromatic prenyltransferase
AR	Androgen receptor
AtaPT	*Aspergillus terreus* aromatic prenyltransferase
BBB	Blood-brain barrier
BLM	Bloom helicase
CHOP	C/EBP homologous protein
*m*-CPBA	*meta*-Chloroperoxybenzoic acid
DBU	1,8-diazabicyclo [5.4.0]undec-7-ene
DCM	Dichloromethane
DCX	Doublecortin X
DDQ	2,3-Dichloro-5,6-dicyano-1,4-benzoquinone
DIA	Differentiation-inducing activity
DMAT	Dimethylallyltryptophan
DMEDA	*N*,*N*′-Dimethylethylenediamine
DMF	*N*,*N*-Dimethylformamide
DPP-IV	Dipeptidyl peptidase-IV
EDDA	Ethylenediamine diacetate
EMC	Endometrial cancer
ER	Endoplasmic reticulum
ERK	Extracellular signal-regulated kinase
fMLP	*N*-Formylmethionyl-leucyl-phenylalanine
GBM	Glioblastoma
GSCs	Glioma stem cells
HIF	Hypoxia-induced factor
HUVEC	Human umbilical vein endothelial cells
ICD	Immunogenic cell death
iNOS	Inducible nitric oxide synthase
IPP	Isopentenyl diphosphate
JNK	c-Jun N-terminal kinase
Keap1	Kelch-like ECH-associated protein 1
LPS	Lipopolysaccharide
MAPK	Mitogen-activated protein kinase
MBC	Minimum bactericidal concentration
MDR	Multidrug resistance
MIC	Minimum inhibitory concentration
MLKL	Mixed lineage kinase domain-like protein
MOE	Methoxyethyl
MOM	Methoxymethyl
MRSA	Methicillin-resistant *Staphylococcus aureus*
MSSA	Methicillin-sensitive *Staphylococcus aureus*
MW	Microwave
NSCLC	Non-small cell lung cancer
Nrf2	Nuclear factor erythroid 2–related factor 2
NF-κB	Nuclear factor kappa-B
NNK	4-(methyl-nitrosamino)-1-(3-pyridyl)-1-butanone
PCNA	Proliferating cell nuclear antigen
PI3K/Akt	Phosphoinositide 3-kinase/Protein kinase B
QSAR	Quantitative structure activity relationship(s)
RIP3	Receptor-interacting protein kinase 3
ROS	Reactive oxygen species
RT	Room temperature
SAR	Structure–activity relationship(s)
SI	Selectivity index
SIRT1	Sirtuin 1
SnPP	Tin protoporphyrin IX
SQR	Sulfide:Quinone oxidoreductase
THF	Tetrahydrofuran
TLR4	Toll-like receptor 4
TOPK	T-lymphokine-activated killer cell-originated protein kinase
TRAIL	Tumour necrosis factor-related apoptosis-inducing ligand
TrxRs	Thioredoxin reductases
*p*TsOH	*para*-Toluenesulfonic acid
TPP	Triphenylphosphine
TPP	Tetraphenylporphyin
T2DM	Type 2 diabetes mellitus
WHO	World Health Organisation
